# Understanding and Improving Risk Assessment After Myocardial Infarction Using Automated Left Ventricular Shape Analysis

**DOI:** 10.1016/j.jcmg.2021.11.027

**Published:** 2022-09

**Authors:** Jorge Corral Acero, Andreas Schuster, Ernesto Zacur, Torben Lange, Thomas Stiermaier, Sören J. Backhaus, Holger Thiele, Alfonso Bueno-Orovio, Pablo Lamata, Ingo Eitel, Vicente Grau

**Affiliations:** a Institute of Biomedical Engineering, Department of Engineering Science, University of Oxford, Oxford, United Kingdom; b University Medical Center Göttingen, Department of Cardiology and Pneumology, Georg-August University, Göttingen, Germany; c German Centre for Cardiovascular Research, Göttingen, Germany; d University Heart Center Lübeck, Medical Clinic II, Cardiology, Angiology, and Intensive Care Medicine, Lübeck, Germany; e University Hospital Schleswig-Holstein, Lübeck, Germany; f German Centre for Cardiovascular Research, Lübeck, Germany; g Heart Center Leipzig at University of Leipzig, Department of Internal Medicine and Cardiology, Leipzig, Germany; h Leipzig Heart Institute, Leipzig, Germany; i Department of Computer Science, University of Oxford, Oxford, United Kingdom; j Department of Biomedical Engineering, King’s College London, London, United Kingdom

**Keywords:** cardiovascular magnetic resonance, acute myocardial infarction, risk assessment, 3D shape analysis, left ventricle, fully automated analysis, AMI, acute myocardial infarction, AUC, area under the receiver-operating characteristic curve, CMR, cardiac magnetic resonance, ED, end-diastole, ES, end-systole, IS, infarct size, LDA, linear discriminant analysis, LV, left ventricle, LVEF, left ventricular ejection fraction, MACE, major adverse cardiac events, MVO, microvascular obstruction, PCA, principal component analysis, SAx, short-axis stacks

## Abstract

**Background:**

Left ventricular ejection fraction (LVEF) and end-systolic volume (ESV) remain the main imaging biomarkers for post-acute myocardial infarction (AMI) risk stratification. However, they are limited to global systolic function and fail to capture functional and anatomical regional abnormalities, hindering their performance in risk stratification.

**Objectives:**

This study aimed to identify novel 3-dimensional (3D) imaging end-systolic (ES) shape and contraction descriptors toward risk-related features and superior prognosis in AMI.

**Methods:**

A multicenter cohort of AMI survivors (n = 1,021; median age 63 years; 74.5% male) who underwent cardiac magnetic resonance (CMR) at a median of 3 days after infarction were considered for this study. The clinical endpoint was the 12-month rate of major adverse cardiac events (MACE; n = 73), consisting of all-cause death, reinfarction, and new congestive heart failure. A fully automated pipeline was developed to segment CMR images, build 3D statistical models of shape and contraction in AMI, and find the 3D patterns related to MACE occurrence.

**Results:**

The novel ES shape markers proved to be superior to ESV (median cross-validated area under the receiver-operating characteristic curve 0.681 [IQR: 0.679-0.684] vs 0.600 [IQR: 0.598-0.602]; *P <* 0.001); and 3D contraction to LVEF (0.716 [IQR: 0.714-0.718] vs 0.681 [IQR: 0.679-0.684]; *P <* 0.001) in MACE occurrence prediction. They also contributed to a significant improvement in a multivariable setting including CMR markers, cardiovascular risk factors, and basic patient characteristics (0.747 [IQR: 0.745-0.749]; *P <* 0.001). Based on these novel 3D descriptors, 3 impairments caused by AMI were identified: global, anterior, and basal, the latter being the most complementary signature to already known predictors.

**Conclusions:**

The quantification of 3D differences in ES shape and contraction, enabled by a fully automated pipeline, improves post-AMI risk prediction and identifies shape and contraction patterns related to MACE occurrence.

Preventive and personalized medicine is crucial to improving both the efficiency and efficacy of health care systems.^1^ Advances have significantly improved the prognosis of acute myocardial infarction (AMI) patients worldwide.^2^ Nevertheless, AMI survivors are at risk of recurrent cardiovascular events, and mortality within 6 months sits at 12%.^3^

Cardiac magnetic resonance (CMR) imaging has proven to be uniquely suitable to assess morphological and functional myocardial alterations, including left ventricular (LV) remodeling, which is central to AMI early prognosis prediction.^4^ LV micromyocardial injury, assessed by means of late gadolinium enhancement CMR and quantified as infarct size (IS) and microvascular obstruction (MVO), has emerged as a robust measure in AMI risk assessment.^4^^,^^5^ Nonetheless, LV macro-function, typically quantified as LV ejection fraction (LVEF), remains the preferred image biomarker in AMI guidelines.^3^^,^^4^ Patients with pumping function deterioration, at higher risk of severe cardiac events, also exhibit an increase in end-systolic volume (ESV), which is reported in several studies as superior to LVEF in predictive power;^6^ and patients with early revascularization and preserved pumping function are characterized by an increase in LV wall thickening in both infarcted and remote regions.^7^

LVEF and ESV are limited to global systolic function and fail to capture functional and anatomical regional abnormalities.^4^^,^^6^^,^^7^ Moreover, LVEF is mainly preserved or only moderately reduced in most AMI survivors, and therefore recurrent adverse events often occur in patients at a theoretical low risk, which further evidences the need of stratification improvements.^8^ To overcome such limitations, unraveling of the global LV remodeling changes at the different regions of the ventricle, ie, the 17-segment American Heart Association model, has been proposed.^9^ High-dimensional 3-dimensional (3D) shape analysis is also used in cardiac research to describe subtle focal changes.^10^

The present study aimed to disentangle ESV and LVEF into 3D shape and contraction with the use of only standard CMR short-axis stacks (SAx) to: 1) gain a better understanding of how 3D remodeling patterns modulate risk and the interplay between macro-remodeling and microdamage; and 2) improve AMI risk stratification. We present a fully automated shape analysis pipeline including state-of-the-art artificial intelligence and 3D meshing and its application on a large multicenter population including ST-segment elevation myocardial infarction (STEMI) and non-STEMI (NSTEMI) patients.

## Methods

### Study population

Our retrospective population consisted of 1,235 AMI survivors^8^ from 2 multicenter randomized trials, AIDA-STEMI (Abciximab Intracoronary Versus Intravenously Drug Application in ST-Elevation Myocardial Infarction; NCT00712101) and TATORT-NSTEMI (Thrombus Aspiration in Thrombus Containing Culprit Lesions in Non–ST-Elevation Myocardial Infarction; NCT01612312).^11^^,^^12^ In both studies, reperfusion therapy with primary percutaneous coronary intervention and postinfarction medical treatment were supplied according to state-of-the-art guideline recommendations.^3^ The size of the study is argued in Supplemental Methods: TRIPOD Reporting (Supplemental Table 1), based on Riley et al.^13^

AIDA-STEMI and TATORT-NSTEMI were registered with ClinicalTrials.gov, were approved by the institute ethics committees, and complied with the Declaration of Helsinki, including written informed consent. Data supporting our findings are available on reasonable request.

### CMR imaging protocol and manual analysis

The same standardized protocol, including electrocardiography-gated balanced steady-state free precession sequences and T1-weighted late gadolinium enhanced images, was followed for all patients with AMI on 1.5-T or 3.0-T clinical scanners.^11^^,^^12^ Horizontal and vertical long-axis views as well as continuous stacks of SAx slices capturing the whole LV were acquired in all sequences. Infarct characteristics, ventricular volumes, and LVEF were determined in sequential SAx by blinded clinicians using certified evaluation software (cmr42, Circle Cardiovascular Imaging).^12^ IS and MVO were assessed in Eitel et al,^12^ applying standard thresholding techniques.

### Study endpoints

The 12-month rate of MACE, consisting of reinfarction, new congestive heart failure, and all-cause death, was the predefined clinical endpoint of the study as detailed previously.^8^^,^^11^^,^^12^ The events were adjudicated by a blinded clinical committee based on the data collected in the study sites. Only 1 contribution per patient to the endpoint composite was considered in case of multiple MACE events per patient (death > reinfarction > congestive heart failure).

### LV fully automated shape analysis

**Figure 1:**
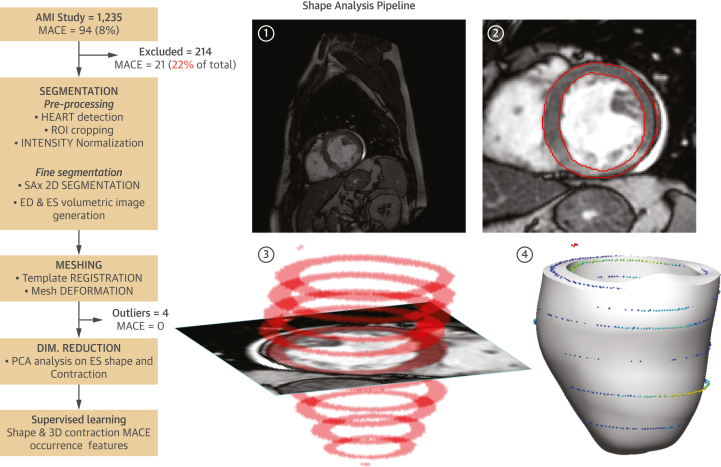
Shape Analysis Pipeline Shape analysis pipeline **(left)** along sample case **(right)**, consisting of the following steps: 1) short-axis stacks (SAx) preprocessing (intensity normalization, heart detection, cropping of region of interest [ROI]); 2) fine segmentation of left ventricular myocardium; 3) volumetric segmentation at end-systole (ES) and end-diastole (ED) phases; and 4) personalized mesh generation. 3-dimensional (3D) contraction is then calculated from the ES and ED meshes, and principal component analysis (PCA) dimensionality reduction is applied on the meshes to facilitate the supervise learning of major adverse cardiac events (MACE) occurrence.

The LV myocardium was segmented in the SAx images, and 2 personalized 3D LV meshes were built for each patient: at end-diastole (ED) and at end-systole (ES). The displacements between corresponding points in them constituted the 3D contraction. 3D shape and contraction descriptors were obtained by unsupervised construction of statistical shape models and used as potential predictive biomarkers. The process (Figure 1) was fully automated.

#### LV segmentation

A 2-step deep learning approach was applied to segment the LV myocardium, based on a UNet architecture with enhanced preprocessing,^14^^,^^15^ that reached the best performance in the 2019 LV Full Quantification Challenge.^15^ ES and ED phases are detected as those of minimal and maximal LV blood pool area at the midventricular slice. Implementations details are provided in Supplemental Methods: LV Segmentation (Supplemental Figure 1).

#### Mesh generation

The construction of 3D meshes from segmentation contours used a solution based on smooth cubic Hermite interpolation, which was shown to be accurate and robust concerning SAx slice misalignment and segmentation errors and fully described in Lamata et al.^16^ In brief, an idealized LV template mesh was fitted to the 3D myocardium mask by combining image registration and mesh projection techniques. Meshing results were evaluated for accuracy using the 3D distance between segmentation contours and mesh surfaces. The anatomical correspondence between meshes was achieved by orienting the LV meshes according to the SAx canonic position.

#### Dimensionality reduction

**Figure 2:**
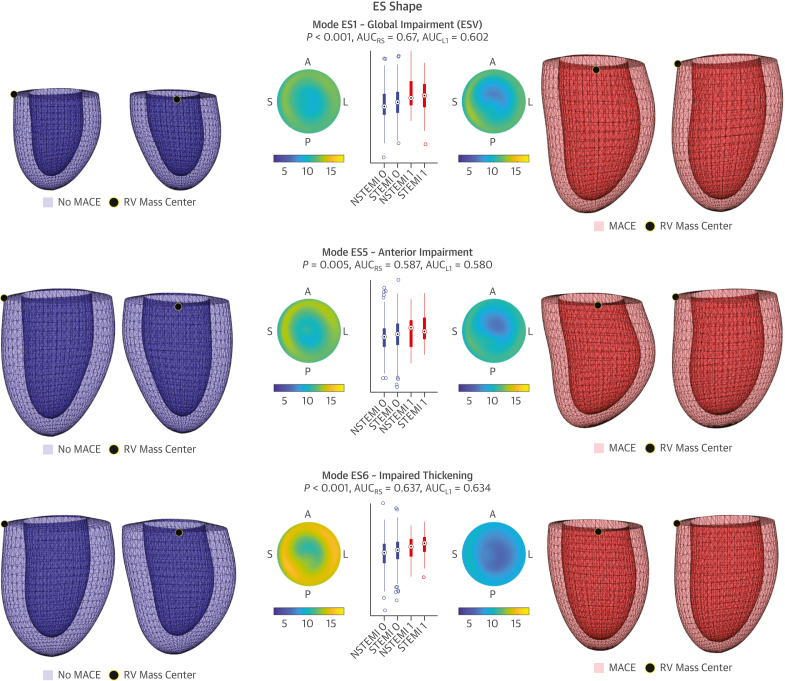

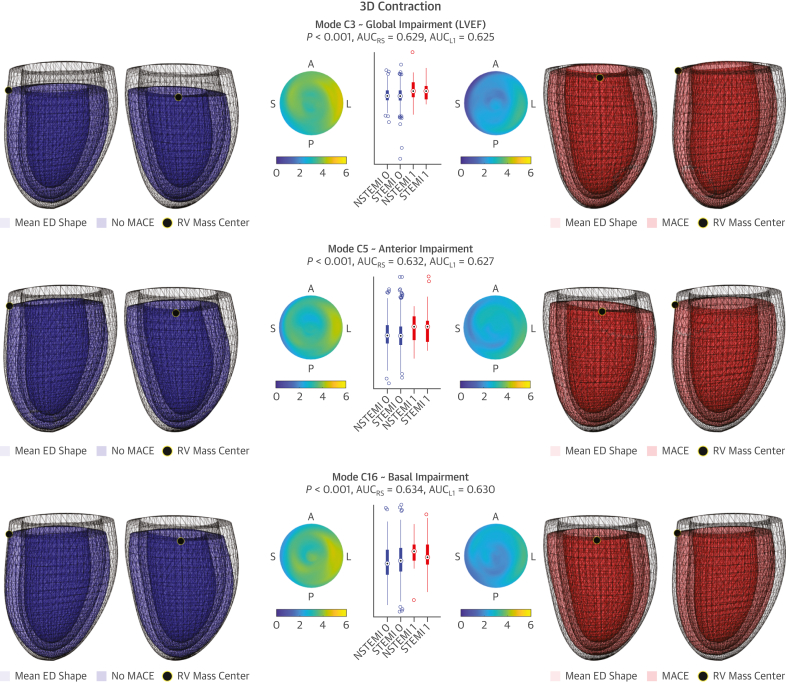
3D LV Shape and Contraction Risk-Related Patterns The 3 main ES shape features (ES1, ES5, ES6) and contraction patterns (C3, C5, C16) related to MACE occurrence are illustrated. Meshes shown in anterior and septal views, and as differential thickness maps (ED-ES thickness) on polar plots of the American Heart Association model. To facilitate comparisons, the contractions are applied on the mean ED shape (reference transparent surface) and visualized as resulting ES shapes. No MACE (**blue**, class 0) and MACE (**red**, class 1) representations correspond to the 5th and 95th percentiles in the LDA direction. *P* values and AUCs shown along MACE and No MACE distributions, further stratified into infarct etiology (ST-segment elevation myocardial infarction [STEMI] and non–ST-segment elevation myocardial infarction [NSTEMI]). AUC = area under the receiver-operating characteristic curve; LDA = linear discriminant analysis; other abbreviations as in Figure 1.

The direct analysis of the 3D meshes, discretized into 2,450 nodes with their corresponding 3D spatial coordinates, is impractical and ill posed. This motivates the use of principal component analysis (PCA) for dimensionality reduction.^10^ In short, PCA inspects the data to find the directions of change that maximize the variance observed in the population. The resulting main directions represent the 3D shape patterns of change with respect to the mean shape, which are called anatomical “PCA modes.” Conceptually, the LV of each subject is decomposed into the mean shape (average ventricle) plus the anatomical modes (shape variations, ie, thickening, scaling, lengthening, etc) times a coefficient. Each of the modes is therefore a continuous variable that accounts for a particular pattern of 3D shape variation, which has a certain value for each patient and becomes the potential biomarker to predict MACE (Figure 2). The formulation details are explained in Supplemental Methods: Dimensionality Reduction (Supplemental Figures 2 to 5).

Three statistical shape models were built with this PCA technique, studying the 3D shape at ED and at ES, and the displacement between them (contraction). PCA coefficients of ED were found to contain no predictive value and are therefore not reported.

### Supervised learning

The relevant PCA modes to predict MACE were found by a stepwise multivariable Fisher linear discriminant analysis (LDA), as detailed below. The search was performed on the ES shape and on the contraction modes needed to reconstruct 95% of the total variance of the population. This selection was further refined with another LDA applied on the combination of ES and contraction-significant modes found in the previous step.

### Evaluation experiments

The predictive value of the novel ES shape and contraction PCA modes was evaluated against standard clinical biomarkers and in combination with them. Conceptually, ES and contraction PCA modes are the 3D extension of the ESV and LVEF biomarkers, and a first test compared them. Multivariable combinations were next investigated, considering only CMR biomarkers and all the clinical variables of the study together.

Comparisons were based on the prediction performance resulting from both a multivariable LDA (binary classification of MACE occurrence at 12 months) and a multivariable Cox analysis (time to MACE). A backward stepwise strategy (unbiased to univariable associations^17^) was followed in both analyses to find intervariable synergies and address collinearity: All variables of interest were initially included, and the less significant in the model iteratively removed, until a 0.05 *P* value threshold was met by all of them. Significance in the LDA was approximated as significance in the generalized linear binomial sigmoid regression model. To account for both specificity and sensitivity and class imbalance, the performance of the resulting configurations was assessed via area under the receiver-operating characteristic curve (AUC) for binary classifications and by concordance index (C-index) for time-dependent curves.

The generality of findings was evaluated by comparison of performance in the resubstitution (apparent performance: learning and testing with the entire cohort) and cross-validation (optimism-corrected performance: 10 cross-fold-validation repeated for 100 random data splits). To further strengthen this generalization, an additional experiment trained the models only on AIDA-STEMI patients and tested them on the TATORT-NSTEMI independent cohort (Supplemental Results: Independent Testing, Supplemental Figure 6). A reproducibility analysis, where the ED and ES frames were removed to test the robustness of the new methodology, is included in Supplemental Results: Reproducibility Analysis (Supplemental Table 2, Supplemental Figures 7 and 8).

### Statistical analysis

The variables of the study (baseline characteristics, cardiovascular risk factors, CMR biomarkers, and significant ES and contraction PCA coefficients) are described according to MACE occurrence. Continuous variables are presented as median (IQR), because they were not normally distributed in a Shapiro-Wilk test except for the modes of variation (which are gaussian distributed by definition). Categoric variables are presented as frequencies and percentages. The MACE and No MACE distributions, as well as the cross-validated AUC distributions, were compared by means of the nonparametric Wilcoxon rank sum test. Univariate Cox regression analyses were also performed. HRs, 95% CIs, and the predictor significance are presented in the results. All analyses were implemented in Matlab R2019b. The study follows the guidelines of the Transparent Reporting of Multivariable Prediction Model for Individual Prognosis or Diagnosis (Supplemental Table 1) and the Prediction Model Risk of Bias Assessment Tool for model development.^17^^,^^18^

## Results

### Patients

A total of 1,021 patients with AMI (STEMI: n = 723; NSTEMI: n = 298) out of the initial 1,235 cohort had both original CMR scans and 12-month follow-up data available and were included in this study (No CMR: n = 126; incomplete protocol: n = 86; no follow-up: n = 2).^8^^,^^11^^,^^12^ They underwent CMR a median of 3 days (IQR 2-4 days) after infarction and presented a total of 73 MACE (congestive heart failure: n = 20; reinfarction: n = 21; death: n = 32).^11^^,^^12^

CMR and baseline clinical characteristics are described in Table 1. As reported previously,^8^^,^^11^^,^^12^ the population was predominantly male, with an overall median age of 63 years, LVEF 50.5%, and IS 13.4% of LV mass, with minor MVO. The MACE subgroup was significantly older, with higher body surface area, a lower percentage of smokers, and greater prevalence of hypertension and diabetes mellitus. They also presented larger ESV, IS, MVO, Killip class on admission, and number of diseased vessels and lower LVEF.

**Table 1:** Baseline Characteristics, Cardiovascular Risk Factors, and CMR Biomarkers

	All Patients	MACE (n = 73)	No MACE (n = 948)	AUC_k_	*P* Value	HR (95% CI)	HR *P V*alue
Age, y	63 (52 to 72)	72 (61 to 77)	63 (52 to 72)	0.659	<0.001	1.80 (1.39-2.32)	<0.001
Sex	753/1,011 (74.5)	46/71 (64.8)	707/940 (75.2)	0.505	0.052	0.81 (0.65-1.00)	0.050
Height, cm	2 (1 to 2)	2 (1 to 3)	1 (1 to 2)	0.597	0.003	0.69 (0.55-0.86)	0.001
Weight, kg	81 (72 to 90)	76 (70 to 86)	82 (73 to 90)	0.568	0.035	0.82 (0.64-1.05)	0.110
Cardiovascular risk factors							
Current smoking	405/935 (43.3)	19/63 (30.2)	386/872 (44.3)	0.522	0.029	0.75 (0.57-0.97)	0.032
Hypertension	716/1,010 (70.9)	61/71 (85.9)	655/939 (69.8)	0.545	0.004	1.53 (1.13-2.08)	0.006
Hyperlipoproteinemia	624/1,005 (62.1)	45/71 (63.4)	579/934 (62.0)	<0.5	0.816	1.03 (0.81-1.30)	0.824
Diabetes mellitus	231/1,010 (22.9)	26/71 (36.6)	205/939 (21.8)	0.526	0.004	1.34 (1.09-1.64)	0.005
Body mass index, kg/m^2^	27.4 (25.0 to 30.4)	27.0 (25.2 to 30.8)	27.4 (25.0 to 30.3)	<0.5	0.959	1.03 (0.82-1.30)	0.773
Body surface area, m^2^	1.95 (1.83 to 2.08)	1.88 (1.76 to 2.00)	1.96 (1.83 to 2.08)	0.593	0.006	0.74 (0.59-0.94)	0.014
Killip class on admission				0.573	<0.001	0.52 (0.41-0.65)	<0.001
1	899/1,011 (88.9)	49/71 (69.0)	850/940 (90.4)				
2	76/1,011 (7.5)	13/71 (18.3)	63/940 (6.7)				
3	20/1,011 (2.0)	4/71 (5.6)	16/940 (1.7)				
4	16/1,011 (1.6)	5/71 (7.0)	11/940 (1.2)				
No. of diseased vessels				0.567	0.003	1.40 (1.12-1.75)	0.003
1	502/1,011 (49.7)	25/71 (35.2)	477/940 (50.7)				
2	310/1,011 (30.7)	23/71 (32.4)	287/940 (30.5)				
3	199/1,011 (19.7)	23/71 (32.4)	176/940 (18.7)				
TIMI flow grade after PCI				<0.5	0.318	0.95 (0.77-1.16)	0.598
0	19/1,011 (1.9)	1/71 (1.4)	18/940 (1.9)				
1	21/1,011 (2.1)	2/71 (2.8)	19/940 (2.0)				
2	78/1,011 (7.7)	8/71 (11.3)	70/940 (7.4)				
3	893/1,011 (88.3)	60/71 (84.5)	833/940 (88.6)				
CMR biomarkers							
LV ESV, mL	70 (53 to 91)	86 (60 to 110)	69 (53 to 90)	0.599	0.004	1.43 (1.18-1.73)	<0.001
LV EDV, mL	144 (117 to 171)	145 (121 to 170)	144 (117 to 172)	<0.5	0.987	1.05 (0.83-1.33)	0.679
LVEF, %	50.5 (43.3 to 57.3)	40.6 (33.1 to 52.2)	50.8 (44.0 to 57.5)	0.683	<0.001	0.80 (0.74-0.87)	<0.001
Infarct size, mL	17.2 (6.4 to 30.2)	24.6 (9.7 to 36.4)	16.7 (6.0 to 29.9)	0.591	0.006	1.29 (1.08-1.53)	0.005
Infarct size (% LV mass)	13.4 (5.4 to 21.8)	20.3 (9.6 to 28.9)	13.1 (5.3 to 21.4)	0.609	0.001	1.44 (1.18-1.76)	<0.001
MVO, mL	0.00 (0.00 to 1.90)	0.40 (0.00 to 3.00)	0.00 (0.00 to 1.80)	0.543	0.060	1.27 (1.11-1.45)	<0.001
MVO (% LV mass)	0.00 (0.00 to 1.39)	0.32 (0.00 to 2.15)	0.00 (0.00 to 1.27)	0.547	0.044	1.26 (1.09-1.46)	0.002
ES shape				0.680			
Mode 1	−5 (−135 to 126)	62 (−61 to 233)	−10 (−136 to 119)		0.002	1.49 (1.18-1.87)	<0.001
Mode 5	0 (−34 to 36)	−11 (−51 to 22)	1 (−33 to 37)		0.014	0.73 (0.58-0.91)	0.006
Mode 6	−3 (−30 to 28)	−21 (−44 to 3)	−2 (−29 to 30)		<0.001	0.64 (0.50-0.81)	<0.001
Contraction displacement				0.716			
Mode 3	−4 (−54 to 62)	49 (−29 to 106)	−6 (−55 to 59)		<0.001	1.70 (1.36-2.12)	<0.001
Mode 5	3 (−37 to 39)	−30 (−57 to 23)	4 (−33 to 40)		<0.001	0.63 (0.51-0.78)	<0.001
Mode 16	1 (−15 to 14)	−9 (−19 to 5)	1 (−14 to 15)		<0.001	0.65 (0.52-0.82)	<0.001

Values are median (IQR) or n/N (%). *P* values calculated between MACE and No MACE groups. HRs presented with 95% CIs and predictor significance. AUC_k_ provides the predictive power of each biomarker, assessed via linear discriminant analysis (median AUC, 10-cross-fold validated, 100 random data splits).

AUC = area under the receiver-operating characteristic curve; CMR = cardiac magnetic resonance; EDV = end-diastolic volume; ESV = end-systolic volume; LV = left ventricular; LVEF = left ventricular ejection fraction; MACE = major adverse cardiac events; MVO = microvascular obstruction; PCI = percutaneous coronary intervention; TIMI = Thrombolysis in Myocardial Infarction.

### Shape analysis

The automated segmentation of the SAx, including all the planes and phases, resulted in median Dice scores of 0.971 (IQR: 0.945-0.981) and 0.975 (IQR: 0.959-0.982) for LV epicardium and endocardium, respectively. The Spearman correlation coefficients (*r*_s_) between the volumes calculated from automated and manual segmentations were 0.916, 0.919, and 0.842 for ESV, EDV, and LVEF, respectively. The median distances between mesh surfaces and contours were 0.981 mm (IQR: 0.806-1.22 mm) for ES and 1.02 mm (IQR: 0.828-1.31 mm) for ED. The *r*_s_ coefficients between the manual volumes and those integrated from the mesh were 0.905, 0.914, and 0.792 for ESV, EDV, and LVEF, respectively. The resulting LVEF calculated from the meshes was negative for 4 patients because of noise in the midventricular segmentation that led to an incorrect phase selection. They were excluded from the statistical analysis (Figure 1).

Ninety-five percent of the variance was captured by the first 13 ES shape and the 21 first contraction modes of variation in PCA analyses. The LDA stepwise analysis on the combination of ES and contraction significant modes converged to ES modes 1, 5, and 6 and contraction modes 3, 5, and 16 (Figure 2, Videos 1 and 2). These modes are interpreted as basal (C16), anterior (ES5, C5), and global impairment, in the form of increased ESV (ES1), decreased LVEF (C3) and impaired thickening (ES6) (see Discussion).

### Endpoint prediction

The automated volumes calculated from the mesh led to a moderate but significant improvement in performance (*P <* 0.001) compared with manual contours (Central Illustration).

**Central Illustration:**
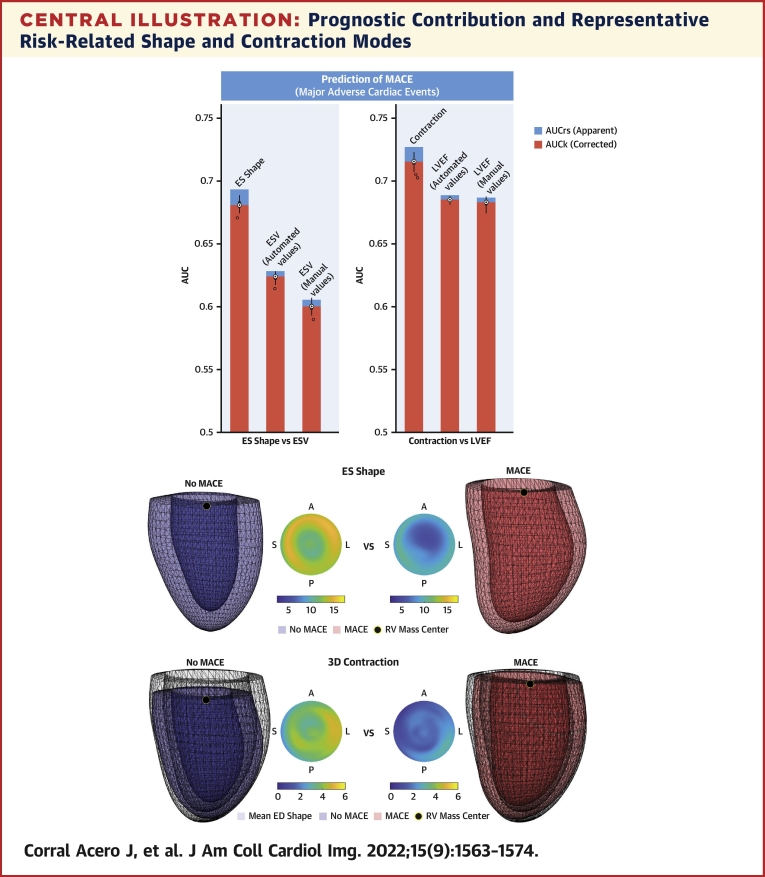
Prognostic Contribution and Representative Risk-Related Shape and Contraction Modes **(Left)** MACE prediction comparison between ESV and LVEF and the proposed 3D disentanglement into ES shape and contraction. Assessment based on AUC resubstitution (AUC_rs_, **blue**) and 10-cross-fold validation (AUC_k_, **orange**), repeated for 100 random data splits **(black distributions)**. All prediction differences were significant (*P <* 0.001); y-axis origin set to 0.5 (random classifier). **(Right)** Anterior views of representative No MACE **(blue)** vs MACE **(red)** ES shape and 3D contraction, and differential thickness maps (ED-ES thickness) in American Heart Association model representation. Contractions are applied on the mean ED shape (transparent surface) and visualized as resulting ES shapes to ease comparisons. 3D = 3-dimensional; AUC = area under the receiver-operating characteristic curve; ED = end-diastole; ES = end-systole; ESV = end-systolic volume; LVEF = left ventricular ejection fraction; MACE = major adverse cardiac events.

The most predictive variables of the study were ES shape and contraction, followed by LVEF, age, IS, and ESV (Table 1). The LDA on ES shape, described as the combination of ES modes 1, 5, and 6, improves the cross-validated AUC (AUC_k_) of endpoint prediction from 0.60 to 0.68 (*P <* 0.001) with respect to ESV; the LDA on 3D contraction, described by the contraction modes 3, 5, and 16, increases from 0.68 to 0.72 (*P <* 0.001) compared with LVEF (Central Illustration).

The combination of the 3D descriptors with only CMR biomarkers (AUC_k_ = 0.738) and including cardiovascular factors and basic patient characteristics (AUC_k_ = 0.747) led to a further improvement compared with the clinical baselines (*P <* 0.001). Similar results were obtained for C-indexes (Table 2). The LDA and Cox analyses converged to the same selection of variables except for a deviation in 1 variable. The differences in performance hold in the independent testing experiment (Supplemental Figure 6). The resulting receiver-operating characteristic curves, Kaplan-Meier estimates, and additional multivariable model experiments are included in Supplemental Figures 9 to 13 and Supplemental Tables 3 to 6.

**Table 2:** Risk Prediction Results

Type	Model	Linear Selection	AUC_k_	AUC_RS_	Cox Selection	C-Index_k_	C-Index_RS_
Clinical baseline	ESV	ESV	0.600 (0.598-0.602)	0.605	ESV	0.611 (0.610-0.612)	0.614
	LVEF	LVEF	0.682 (0.681-0.685)	0.687	LVEF	0.669 (0.668-0.670)	0.671
3D analysis	ES shape	ES1, ES5, ES6	0.681 (0.679-0.684)	0.693	ES1, ES5, ES6	0.667 (0.665-0.669)	0.677
	3D contraction	C3, C5, C16	0.716 (0.714-0.718)	0.727	C3, C5, C16	0.700 (0.698-0.702)	0.709
Multivariable	CMR	ESV, EDV, C5, C16	0.738 (0.736-0.740)	0.750	ESV, EDV, C5, C16	0.728 (0.727-0.730)	0.736
	All	ESV, EDV, age, Killip,^a^ C5, C16	0.747 (0.745-0.749)	0.766	ESV, EDV, Age, C5, C16	0.741 (0.739-0.744)	0.753

Comparative analysis (linear discriminant analysis and Cox) of clinical baseline, 3D descriptors, and multivariable models with only CMR vs all clinical variables. ESV, EDV, and LVEF calculated from automated volumes (results with manual volumes presented in Supplemental Table 6). Final selections of variables are reported along with their predictive performances, expressed as AUC and C-index, under cross-validation (k = 10) and resubstitution (RS). AUC_k_ and C-index_k_ are presented as median (IQR). All differences were statistically significant (*P <* 0.001).

3D = 3-dimensional; other abbreviations as in Table 1.

a Killip class on admission.

## Discussion

In this study, we present the first large-sized multicenter CMR study that comprehensively analyzes the LV 3D shape and contraction after AMI for risk assessment. We successfully 1) combined AI and computational anatomy technologies to develop a fully automated pipeline that segments the cine CMR SAx stack and identifies the 3D signatures of AMI that predict risks along the first year of follow-up; 2) identified these LV shape and contraction macrofeatures related to AMI prognosis and built a 3D reference atlas from more than 1,000 AMI patients; and 3) proved that a 3D enhancement of ESV and LVEF not only outperforms the standalone versions, but also contributes to a significant overall risk prediction improvement in a multivariable setting including CMR markers, cardiovascular risk factors, and basic patient characteristics.

### AMI under the lens of automated shape analysis

Our automated method takes a CMR stack, segments the LV, and summarizes its shape and contraction related to AMI into 6 biomarkers. It reduces the time of the analysis to seconds, removing the burden of manual segmentations. The mesh-fitting step^16^ ensures 3D-spatial consistency, smoothing the 2D segmentations (ie, misalignment, segmentation errors) and leading to more accurate volumes. This explains the improvement in MACE prediction driven by the automated volumes compared with the manual ones (Central Illustration).

The method, which runs on a standard laptop, is fully deterministic: Given a scan, it always returns the same volumes and scores. This removes intra- and interobserver variability, producing a robust diagnosis. The large size of the cohort, the small gap between resubstitution and cross-validation metrics, and the narrow variance for the 100 random data splits suggest the robustness of the method for MACE prediction. The independent testing experiment confirmed the generality of the findings (Supplemental Figure 6). This was further supported by the reproducibility analysis studying the effect of deleting the ED and ES frames, which highlights the robustness of the automated volumes (EDV: *R*^2^ = 0.987; ESV: *R*^2^ = 0.990) and confirms a good reproducibility of the method (multivariable: *R*^2^ = 0.901) (Supplemental Table 2, Supplemental Figures 7 and 8), which is similar to those of CMR state-of-art interstudy reproducibility studies.^19^

As a result of the automated shape analysis, we have built a 3D LV reference atlas from more than 1,000 patients with AMI that captures the average shape and contraction after infarction alongside the main variations (PCA modes). These data and the resulting risk models, which have been made publicly available,^20^ will allow further AMI shape studies, computer mechanistic simulations, or synthetic patient data generation for training algorithms or educational use. The segmenter, specifically modified to cope with different protocols and scanners (as described by Corral Acero et al^21^), and the meshing pipeline will be likewise available, opening the scope to other cardiac diseases and applications.

### Impact and interpretability of the novel methodology

LVEF and ESV are the most established markers for postinfarction risk assessment. Although there are data suggesting that these parameters can be fully automatically determined with the use of commercially available software,^22^ our proposed methodology is further capable of disentangling them into their enhanced 3D versions. It is expected that proposed 3D biomarkers capture more information and achieve additional prognostic value compared with their unidimensional versions, as shown in the Central Illustration (the implication of these AUC improvements is illustrated in Supplemental Figure 12). Indeed, although the main motivation of the study was not prediction performance but rather understanding how the 3D features modulate risk, the prognostic value of these 3D biomarkers was superior to any of the variables of the study (Table 1) and even to the combination of all of the tissue CMR biomarkers (Supplemental Figure 11).

Furthermore, these 3D patterns are complementary to the other variables included in the study (Table 1), as evidenced by their significant contribution in any of the considered multivariable approaches (Table 2), unlike, eg, IS, which is predictive according to the univariate analysis but does not significantly add value to the multivariable models^8^ and therefore was not included in the final selection of variables. These results demonstrate the prognostic value of the novel 3D metrics in AMI postinfarction risk assessment, significantly contributing to multivariable models that improve MACE prediction from 0.68 to 0.75 (cross-validated AUC) compared with LVEF, the clinical baseline (Table 2).

The severity of the infarct is associated with morphological and functional alterations, and certain acute changes have proven to be important to AMI prognosis, but their interplay in modulating MACE occurrence remains unsolved.^4^^,^^6^^,^^7^ In this study, we identified 3 ES shape variations and 3 contraction patterns relevant to MACE (Figure 2, Videos 1 and 2), that suggest 3 possible impairments caused by AMI: global (C3, ES1, ES6), anterior (C5, ES5), and basal (C16). Because the ED shape was not predictive, the changes in morphology seen in the ES modes should be interpreted as functional and not remodeling. These novel biomarkers are consistent with the predominant LV remodeling descriptors according to the literature: ES1 is strongly correlated with ESV (*r*_s_ = 0.868), C3 with LVEF (*r*_s_ = −0.563), and ES6 with ES myocardial thickness.^3^^,^^6^^,^^7^ Indeed, any of the prognostic contraction modes (C3, C5, C16) captures some degree of the latter thickening impairment. ES5 and C5 are alternative (ie, not correlated) manifestations of anterior impairment, where the posterior wall pulls the weakened anterior wall and causes a shift in the LV vertical axis. This suggests a perfusion impairment in the left anterior descending (LAD) artery, in agreement with Ortiz-Pérez et al.^9^ In addition, C16 points to basal impairment as an additional risk factor (Supplemental Figures 14 to 17, where differences are highlighted), which could be caused by twist differences caused by circumferential fiber damage (resulting in larger basal diameters), in contrast to the endocardial vertical and fiber injuries that lead to decreased LVEF (elongation and wall thickening, respectively). C16, a basal contraction impairment, is the signature most complementary to already known predictors.

The derived risk score model, however, does not include the contraction patterns C2 and C14, which could be interpreted as related to injury in the left circumflex and right coronary artery^9^ (Supplemental Figures 14 to 16). These patterns were not significant in the stepwise analysis, suggesting that they could be implicitly described by linear combinations of the selected MACE signatures.

Myocardial tissue death is central to pumping ability and prognosis, but because of hemodynamic reflexes, quantifying its contribution to acute remodeling and dysfunction is not straightforward.^23^ There is an interplay between myocardial 3D shape changes and microdamage (IS, MVO) that we detected in our data by the correlation of ES1 with IS (*r*_s_ = 0.461) and MVO (*r*_s_ = 0.345). Risk-related shape and contraction variations (except for C16) are also significant in stratifying IS and MVO into low vs high myocardial damage, using the median values of the AMI population as threshold (Table 1, Supplemental Results: Modes Correlation Analysis, Supplemental Figures 18 to 21). This explains why IS and MVO are not included in the multivariable risk assessment models: The myocardial damage information is implicit to the identified shape and contraction modes.

The analysis of endpoints prediction stratifying by sex, infarct etiology, and LVEF is discussed in Supplemental Results: Subgroup Analysis (Supplemental Figure 17).

### Toward clinical translation

CMR-based risk models have not yet found their role in clinical practice despite multiple trials showing their incremental prognostic information in AMI management.^8^ This is partly explained by the more common availability of echocardiography, the complexity of CMR multiparameter models, and the requirement for significant manual interaction.^3^^,^^8^ Nonetheless, CMR availability has significantly increased in recent years, CMR postinfarction protocols have been shortened, and complex CMR prognostic markers can now be combined into simple risk score models,^8^ as we have shown. Our work further contributes to facilitating the adoption of CMR postinfarction risk management in clinical routine by removing the burden of manual segmentation, along with the intraobserver and interobserver variability, and boosting MACE prediction with the use of enhanced 3D CMR metrics. Although further trials should be considered to validate the findings of this study, the scientific basis to assume prognosis improvement is solid.

### Study limitations

Patients were imaged within 10 days after infarction in the absence of optimal postinfarction CMR imaging time recommendations.^8^ The effect of this postinfarction imaging time on the proposed 3D modes of variations has not been assessed. It is, however, hypothesized that we were capturing acute injury, whose extent and phenotype can predict outcome, and therefore long-term myocardial adaptations. The main objective of this work was to evaluate this hypothesis in a clinical cohort, and not to optimize MACE prediction. For the latter, advanced nonlinear classifiers could be explored. The additional prognostic information of later chronic remodeling^24^ could be straightforwardly incorporated in our pipeline (eg, scan at 3 months).

The study focuses on only 2 instances of the cardiac cycle (ED and ES). This enables for fair and direct comparisons between traditional metrics (ESV, LVEF) and their 3D versions. Future work will explore the time dimension and benefit from automated segmentations and 3D reconstruction across the cardiac cycle, presumably finding an even superior marker. The temporal CMR strains, reported to be prognostic in postinfarction management,^8^ should then be considered.

The PCA geometric features are limited to the gaussian assumption, yet they satisfactorily cover the observed variability in the cohort. Although other approaches^25^ could be considered for dimensionality reduction, they usually come at the cost of interpretability. The PCA selection of features is unsupervised, not biased to outcomes prediction but entirely based on variance, which intrinsically avoids noise and eases interpretability.

CMR long-axis views were excluded from the 3D shape regression for the sake of simplicity, which, although it was not evidenced in our results, may result in shape-lengthening noise. Finally, the results are limited to patients with AMI who can undergo a CMR scan.

## Conclusions

This multicenter CMR study evidences the prognostic value of novel LV 3D shape and contraction metrics in AMI risk assessment. Besides, it further proves the feasibility of fully automated CMR analysis and the significance of multivariable CMR models in AMI stratification.

Perspectives **COMPETENCY IN MEDICAL KNOWLEDGE:** This large-size multicenter CMR study identifies the 3D LV patterns related to postinfarction risk and proves their superior prognostic information compared with the most-established predictors, EF and ESV. Moreover, it highlights the value of multivariable CMR models in AMI stratification and further supports the feasibility of fully automated CMR analysis.

**TRANSLATIONAL OUTLOOK:** The study demonstrates the potential of 3D computational models to improve myocardial infarction management. This is attained by building a refined understanding on the functional and structural interplay that modulates risk, and by bringing the robustness of state-of-the-art tools, which ease the clinical workflow and pave the road toward multivariable models and CMR imaging use. To further accelerate this vision and facilitate future contributions, the 3D shape and contraction atlases and shape models resulting from the study have been made publicly available.

## Funding Support and Author Disclosures

This work was supported by the EU’s Horizon 2020 research and innovation program under Marie Sklodowska-Curie (ga 764738), the German Center for Cardiovascular Research, the British Heart Foundation (PG/16/75/32383, FS/17/22/32644), and the Wellcome Trust (209450/Z/17). The authors have reported that they have no relationships relevant to the contents of this paper to disclose.

## Appendix

Supplemental Data

Supplemental Video I Interpretability of the ES shape patterns found to be related to AMI prognosis.2

Supplemental Video II Interpretability of the 3D contraction patterns found to be related to AMI prognosis.3
