# Supplementary material for: Understanding and Improving Risk Assessment After Myocardial Infarction Using Automated Left Ventricular Shape Analysis
Source: JACC Cardiovasc Imaging. 2022 Sep;15(9):1563–74. doi: 10.1016/j.jcmg.2021.11.027 (PMC9444994; doi:10.1016/j.jcmg.2021.11.027)
Supplement: Supplemental Data [file mmc1.pdf]

SUPPLEMENTAL METHODS: TRIPOD Reporting

Supplemental Table 1. TRIPOD Checklist

DESCRIPTION	ITEM	CHECKLIST	MANUSCRIPT CHECK
**Title & abstract**			
Title	1	Identify the study as developing and/or validating a multivariable prediction model, the target population, and the outcome to be predicted	Title
Abstract	2	Provide a summary of objectives, study design, setting, participants, sample size, predictors, outcome, statistical analysis, results, and conclusions	Abstract
**Introduction**			
Background and objectives	3a	Explain the medical context (including whether diagnostic or prognostic) and rationale for developing or validating the multivariable prediction model, including references to existing models	Introduction (par.1-3)
	3b	Specify the objectives, including whether the study describes the development or validation of the model or both	Introduction (par 3)
**Methods**			
Source of data	4a	Describe the study design or source of data (e.g., randomized trial, cohort, or registry data), separately for the development and validation data sets, if applicable	Methods (Study population)
	4b	Specify the key study dates, including start of accrual; end of accrual; and, if applicable, end of follow-up	Methods (Study population and study endpoints)
Participants	5a	Specify key elements of the study setting (e.g., primary care, secondary care, general population) including number and location of centers	Methods (Study population)
	5b	Describe eligibility criteria for participants	
	5c	Give details of treatments received, if relevant	
Outcome	6a	Clearly define the outcome that is predicted by the prediction model, including how and when assessed	Methods (Study endpoints)
	6b	Report any actions to blind assessment of the outcome to be predicted	
Predictors	7a	Clearly define all predictors used in developing the multivariable prediction model, including how and when they were measured	Methods (Evaluation experiments and statistical analysis), Table 1
	7b	Report any actions to blind assessment of predictors for the outcome and other predictors	
Sample size	8	Explain how the study size was arrived at	*
Missing data	9	Describe how missing data were handled (e.g., complete-case analysis, single imputation, multiple imputation) with details of any imputation method	Fig.1 and Results (Patients)
Statistical analysis methods	10a	Describe how predictors were handled in the analyses	Methods (Experiments, supervised learning and statistical analysis)
	10b	Specify type of model, all model-building procedures (including any predictor selection), and method for internal validation	
	10d	Specify all measures used to assess model performance and, if relevant, to compare multiple models	
Risk groups	11	Provide details on how risk groups were created, if done	Methods (Study endpoints)
**Results**			
Participants	13a	Describe the flow of participants through the study, including the number of participants with and without the outcome and, if applicable, a summary of the follow-up time. A diagram may be helpful	Table 1, Fig.1 and Results (Patients)
	13b	Describe the characteristics of the participants (basic demographics, clinical features, available predictors), including the number of participants with missing data for predictors and outcome	
Model development	14a	Specify the number of participants and outcome events in each analysis	Table 1, Fig.1 and Results (Patients)
	14b	If done, report the unadjusted association between each candidate predictor and outcome	
Model specification	15a	Present the full prediction model to allow predictions for individuals (i.e., all regression coefficients, and model intercept or baseline survival at a given time point)	Results and Supplemental Material III
	15b	Explain how to use the prediction model	
Performance	16	Report performance measures (with CIs) for the prediction model	Results and Supplemental Material III

Continued

**Discussion**			
Limitations	18	Discuss any limitations of the study (such as nonrepresentative sample, few events per predictor, missing data)	Discussion (limitation)
Interpretation	19b	Give an overall interpretation of the results, considering objectives, limitations, results from similar studies, and other relevant evidence	Discussion
Implications	20	Discuss the potential clinical use of the model and implications for future research	Discussion (towards clinical translation)
**Other information**			
Supplementary information	21	Provide information about the availability of supplementary resources, such as study protocol, Web calculator, and data sets	Supplemental Material
Funding	22	Give the source of funding and the role of the funders for the present study	Funding

Transparent Reporting of a multivariable prediction model for Individual Prognosis Or Diagnosis (TRIPOD) checklist, adapted from (17). Only model development items are included. (*) The size of the study is usually determined based on the variables of interest and their standard deviation. The proposed study cannot benefit from this criterion since the variables (modes of variation) were not defined *a priori*. However, aiming at a margin of error of ≤0.05 in the overall outcome proportion estimate, a mean absolute prediction error of 0.05, a desired shrinkage ≤10% and conservative anticipated Cox-Snell R squared statistic of 0.2, and given the approximate MACE incidence of 7% and the number of predictors indicated in the manuscript, the actual size of the study is superior to the trial target size retrospectively determined by any of the 4 proposed calculation methods explained in Riley et al. (13). Par. indicates paragraph.

SUPPLEMENTAL METHODS: LV Segmentation

A 2-step state-of-art approach was applied to segment the LV endo- and epicardium, from which the LV myocardium can be estimated (14,15):

- In the first step, pre-processing, the images are re-oriented, cropped and normalized. Considering the mid-ventricular SAx slice as reference, the first neural network (NN) detects the position of the heart and defines a region of interest (ROI) of 139.7x139.7 mm centered in the LV. Based on these LV centroid and ROI, the LV is aligned to a canonical position by a rigid registration of the same SAx reference slice to the atlas built in (14). The SAx slices are then cropped accordingly, and the intensities are normalized.
- The second step, fine segmentation, applies another NN to the pre-processed images to regress the enhanced LV segmentation. A final postprocessing is applied to binarize the segmentation predictions and improve the segmentation quality.

A cohort of 100 patients of the study, manually segmented with random resolutions and endpoints, was used to train the 2 NNs, following a 5-fold cross-validation strategy and using the same training-validation-testing split ratio as in (14). Translation, rotation and flipping were used for augmentation. Architectures and implementation are detailed in (14,15). Segmentation performance assessment is based on endocardium and epicardium gold standard Dice scores. This metric accounts for the overlap between manual segmentation and automated prediction and varies between 0 and 1, with 1 corresponding to a perfect match. Supplemental Figure 1 provides a visual sample of the segmentation results.

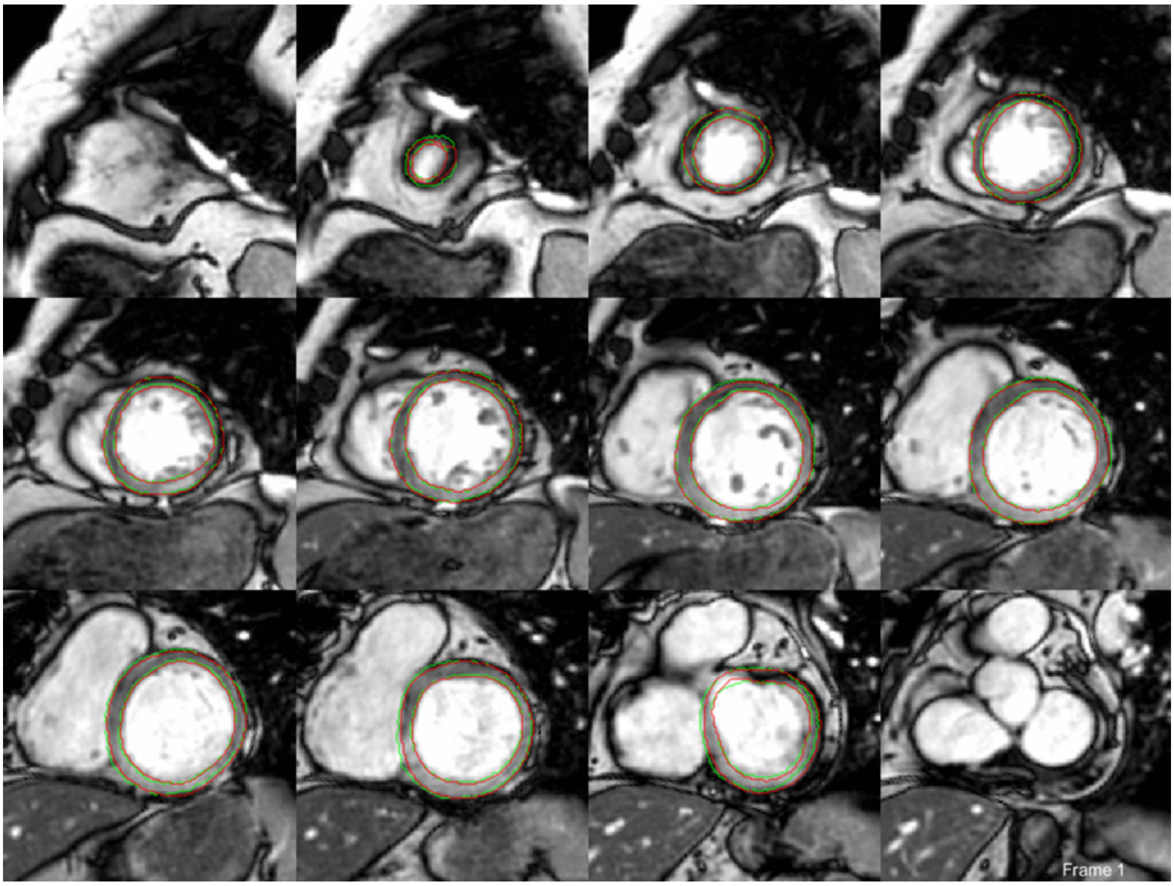

Supplemental Figure 1 – Segmentation results of a representative patient (median Dice) at ED. The green contours correspond to the LV reference segmentation (manual segmentation); and the red contours, to the prediction results based on our proposed 2-step deep learning approach.

SUPPLEMENTAL METHODS: Dimensionality Reduction

As explained in the manuscript, the personalized LV shape of each patient is obtained by fitting (deforming) a template mesh to the myocardial segmentation of the patient. The LV shape of the patient is therefore described by the resulting 3D position (x, y and z cartesian coordinates) of the 2,450 nodes that conform the mesh template, that is, 7,350 variables (nodes × coordinates), which take a certain different value per patient. PCA, the dimensionality reduction technique applied in this work, is able to encode the information contained in these 7,350 variables (shape variability) into a few variables, the anatomical PCA modes, that describe the main shape variations. Mathematically, this is done by finding the vector space whose basis are the orthogonal directions that maximize the variance of the data, and subsequently projecting the data in this new space (See Supplemental Figure 2). These directions that maximize the variance are, precisely, the PCA modes of variation that represent the way in which the shape varies in the population (i.e. thickening, scaling, lengthening, etc.). As illustrated in Supplemental Figure 3, if we move in the direction of a mode, we can see how the mean shape deforms in its specific way. This allows describing each 3D mesh as a mean shape plus the amount of shape change (PCA coefficients) encoded by each linear anatomical mode (See Supplemental Figure 4):

$$Shape = \varphi_0 + \sum_i \omega_i \varphi_i,$$

where φ_0 represents the mean, φ_i the anatomical PCA modes, and ω_i their respective PCA coefficients. Each of the modes, i, is therefore a continuous variable, that accounts for a particular shape variation, which has a certain value for each patient, ω_i, and whose MACE predictive power can be analyzed. In other words, we can investigate which shape features (modes) are related to AMI prognosis.

The modes are sorted in descending order of importance according to the amount of variability of the population that they explain (i.e. in our results 42.6% variability is described by ES mode 1; 19.2%, by ES2; etc.). As we progressively incorporate modes, the shape reconstruction improves to the point that with only a few modes we can accurately approximate any shape (i.e. 95% of ES shape variance described by the first 12 modes), and hence the dimensionality reduction is achieved (See Supplemental Figure 5).

The same principles, herein illustrated for ES shape, apply to contraction.

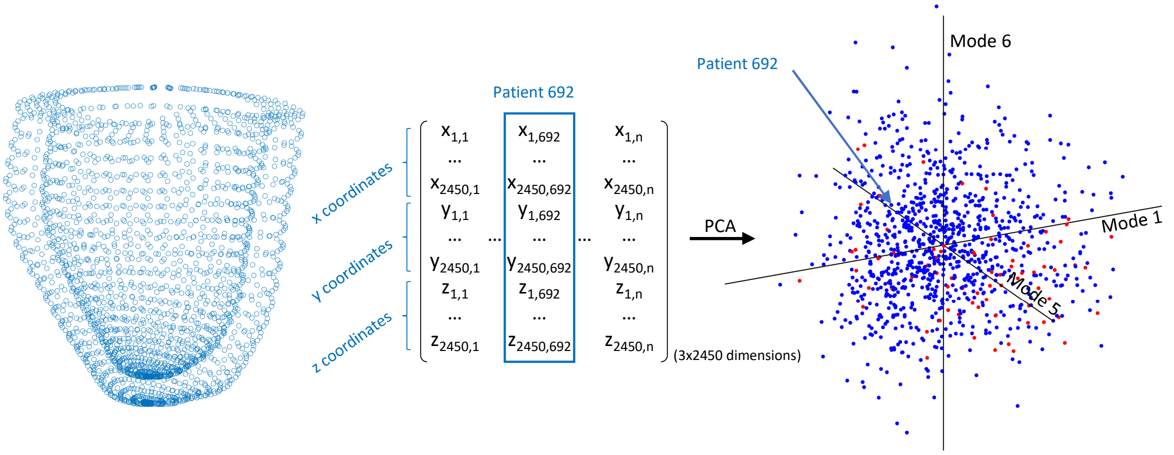

Supplemental Figure 2 – **LEFT**: 3D spatial representation of the 2,450 mesh nodes for patient 692. **MIDDLE**: The 7,350 variables that describe the patient mesh are sorted in columns, one per patient, to apply PCA. **RIGHT**: Projection of the patients in the PCA directions that maximize MACE (red) vs No MACE (blue) differences, i.e. modes 1, 5 and 6. We reduced the number of variables from 7,350 to 3 per patient.

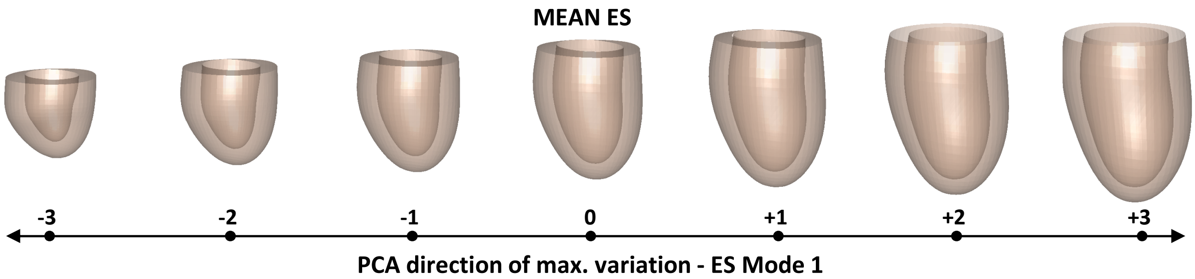

Supplemental Figure 3 – The PCA direction of maximum variability corresponds to ES mode 1. In our results, the shape variation that it encodes is interpreted as mainly scaling (related to ESV, as described in the main manuscript), although some other changes in thickness and morphology can be appreciated. As we move along the ES mode 1 direction, the mean ES shape is reduced (negative ω_1) or enlarged (positive ω_1), proportionally to the value of its PCA coefficient ω_1.

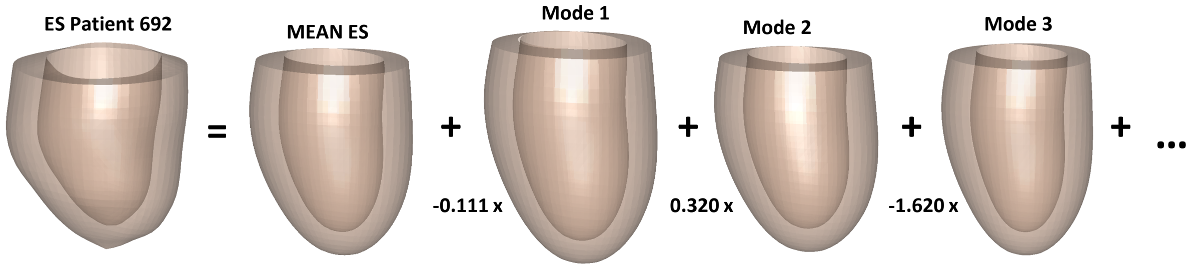

Supplemental Figure 4 – Following PCA application, each LV is decomposed into the mean shape (average LV) plus the anatomical modes (shape variations, illustrated here as mean plus a mode representation) times the corresponding PCA coefficient. The figure illustrates the decomposition of patient 692.

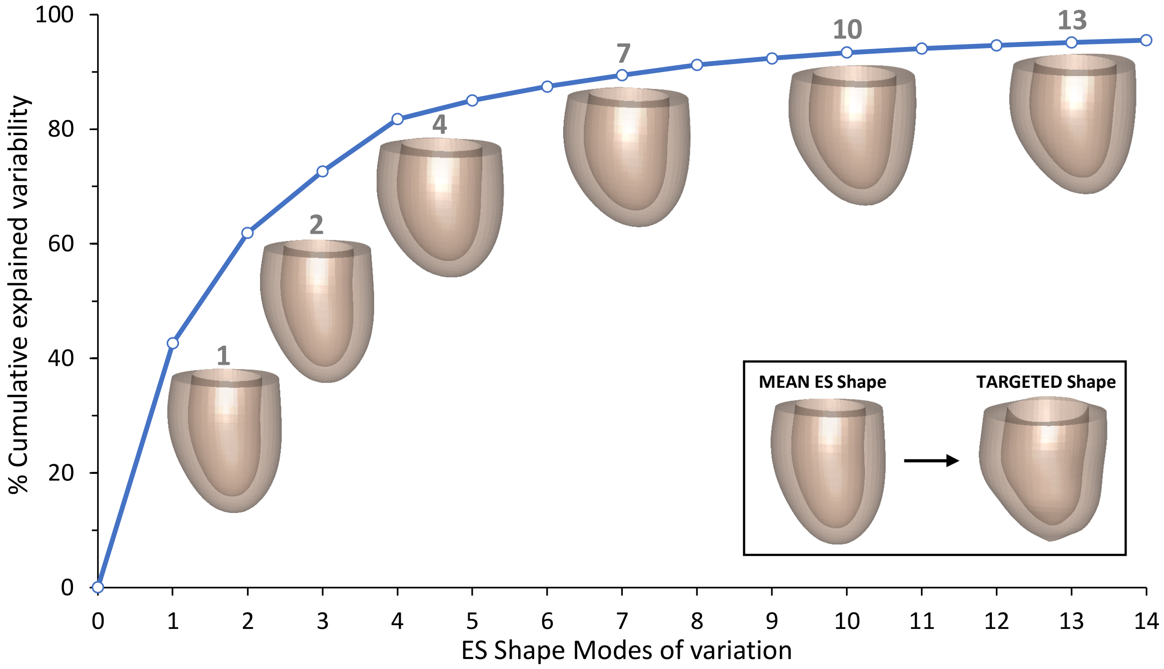

Supplemental Figure 5 – Cumulative % of the population variance explained by the modes. As we incorporate more modes, we account for more variance and we accurately approximate the target shape (Patient 692). The first few modes account for the majority of the variance, and as we move to latter modes the improvement in reconstruction is smaller (i.e. mode 10 vs mode 13).

SUPPLEMENTAL RESULTS: Independent Testing

Given the limited number of MACE events (class imbalance), the split of the cohort into fixed training and testing datasets would affect the statistical strength of the results. Cross-fold validation is therefore preferred and argued in the TRIPOD and PROBAST guidelines to be more robust than the traditional training-testing split, ensuring fair performance and conclusions reporting (17,18). In the *k*-fold cross-validation, the method deployed in this work, the data is randomly partitioned into *k* roughly equal subsets (i.e. folds). Iteratively, each of the *k* subsets is retained (completely unseen) for testing the model, and the remaining *k*-1 subsets are used as training data. The *k* results are then averaged to produce a performance estimation, which reduces noise variability. In addition, the *k*-fold cross-validation process can be repeated for a number of random data splits (e.g. a hundred) to estimate the variance of the model performance.

For completeness, the experiments are repeated, and the performance is evaluated considering an independent testing cohort. The population is split into training-testing based on the two independent randomized trials that compose the study (see Methods). Thus, the developed models are trained from scratch with patients from the AIDA-STEMI trial (n=723) and are evaluated on the TATORT-NSTEMI independent cohort (n=298). Note that the predictor selection is not repeated since this experiment is meant to assess generality and robustness of deployed models and not to develop new ones. The resubstitution and cross-fold validated AUC scores resulting from the training set, along with the final performance on the independent testing set, are illustrated in Supplemental Figure 6. The performance trends reported in the main manuscript (ES shape>ESV; 3D contraction>LVEF; Multivariable models>>Clinical baselines) hold.

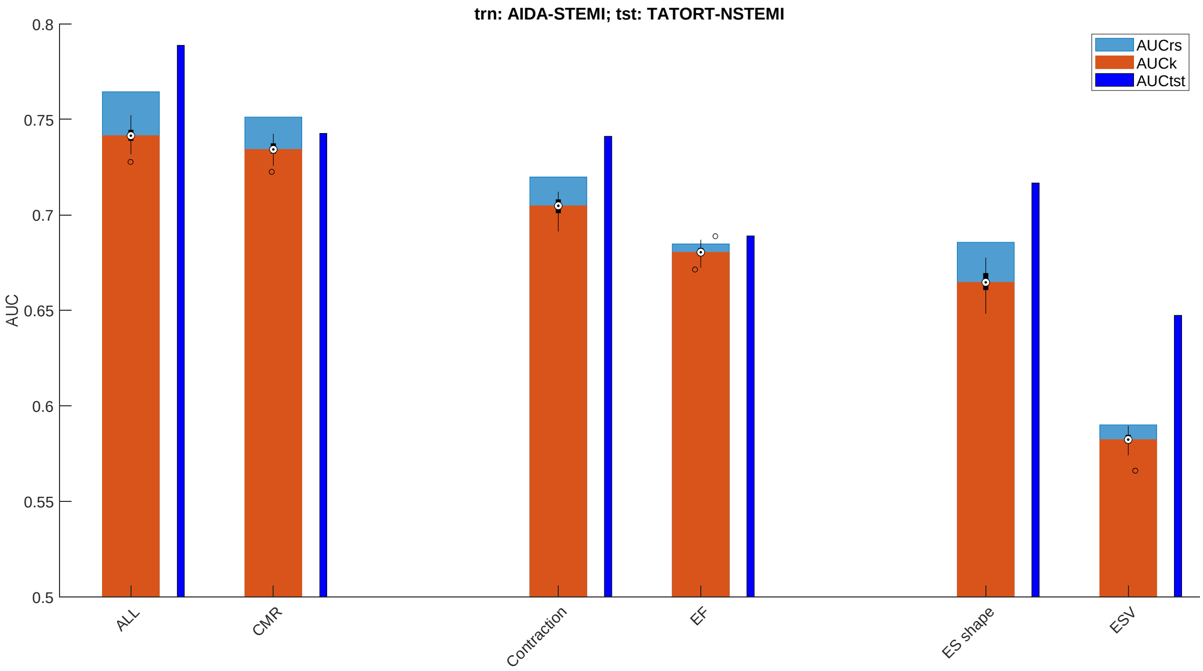

Supplemental Figure 6 –MACE prediction results of the clinical baselines, 3D descriptors and multivariable models, including only CMR biomarkers (CMR) and all the variables of the study (ALL), on the independent testing validation. Patients are split into training (trn, n=723) and testing (tst, n=298) according to the two randomized trials, AIDA-STEMI and TATORT-NSTEMI. The AUC re-substitution (AUCrs, blue) and AUC 10-cross-fold validated (AUCk, orange), repeated for a hundred random data splits (dark distributions) resulting from the training set are reported. Likewise, the final testing AUC (AUCtst, purple), is illustrated.

While these results strengthen the generality of the findings, it can be argued that this experiment is not strictly equivalent to considering a new additional cohort since the TATORT-NSTEMI patients were indirectly used in the predictor selection. The size of the study, the intrinsic design of the methods and the reproducibility analysis further argue for the robustness of the findings (see Discussion).

SUPPLEMENTAL RESULTS: Reproducibility Analysis

In the absence of a patient rescanning protocol in the MRI study, we assess the reproducibility of the method by removing the ED and ES frames of the patients' CMR scans. We thus find an alternative pair of ED and ES frames, those with the second largest and smallest volumes in the original sequence, respectively. The different frames selected emulate a new acquisition, with the same orientation and patient status, but with the bias caused by the removal of the true ES and ED frames.

The volumes and meshes at ES and the 3D contractions (i.e. ES shape – ED shape) resulting from this reproducibility analysis are projected using the original statistical PCA and LDA models, finding the second observation of the proposed biomarkers (ES1, ES3, ES6, C3, C5, C16). Both conventional (EDV, ESV and EF) and proposed biomarkers are regressed to the original observation (See Supplemental Figure 7), and the predictive power of the second observation is assessed applying the models previously deployed based on the originals (See Supplemental Figure 8) without any further training.

The results show a good robustness of the method in the above described drastic scenario. ESV, EDV and ES shape are calculated from a single frame; as such, their reproducibility is superior to LVEF and 3D contraction, which relay on two segmentations. While the robustness of ES shape ($R = 0.980$) is comparable to ESV and EDV ($R = 0.995$ and $R = 0.994$, respectively), the reproducibility of 3D contraction ($R = 0.864$) decreases with respect to LVEF ($R = 0.967$). This could be explained since 3D contraction encodes mode 16, and reproducibility decreases with the higher order modes as one would expect. That is, the higher level of detail we capture, the larger the impact of noise.

Even though the proposed variables that encode contraction variations are more sensitive to noise than LVEF or ESV, their MACE predictive performance is barely compromised. The novel 3D descriptors remain significantly superior, both considering stand-alone or multivariable models (See Supplemental Figure 8), and by considering the combined score with all or with the variables that have been reproduced (i.e. partial in Supplemental Figure 7). Finally, it is worth highlighting the extraordinary reproducibility of the automated volumes ($R > 0.99$).

The intraclass correlation coefficients (one-way random, single measures) and the Spearman correlation coefficients results, shown in Supplemental Table 2, further support the above discussed. Coefficients of variation (within-subject standard deviation method) are also reported for ESV, EDV and LVEF.

Supplemental Table 2 – Reproducibility analysis results

DESCRIPTION	DIFFERENCE	R	r_s	CoV (%)	ICC
ESV, mL	0.227 (3.324)	0.995	0.993	2.824	0.995
EDV, mL	0.168 (4.654)	0.994	0.992	2.075	0.994
EF	0.000 (0.027)	0.967	0.967	3.949	0.967
C3	-	0.986	0.985	-	0.986
C5	-	0.942	0.943	-	0.941
C16	-	0.973	0.969	-	0.973
ES1	-	0.892	0.869	-	0.891
ES5	-	0.840	0.829	-	0.837
ES6	-	0.832	0.830	-	0.829
ES shape	-	0.980	0.977	-	0.980
Contraction	-	0.864	0.849	-	0.862
Multivariable ALL	-	0.952	0.936	-	0.952
Multivariable ALL (partial)	-	0.910	0.895	-	0.910

Reproducibility analysis results reported as: difference, expressed as mean error (SD); linear correlation coefficient, R; Spearman correlation coefficient, r_s; coefficient of variation, CoV; and intraclass correlation coefficient, ICC. Differences and CoV are not reported for the LDA variables (normalized) and the modes of variation (centered at zero by definition). Multivariable ALL indicates scores of the resulting LDA model including all the variables of the study on top of the proposed 3D descriptors; Multivariable ALL (partial), partial scores of the Multivariable ALL model excluding the invariant markers in the reproducibility analysis (i.e. Age, etc.).

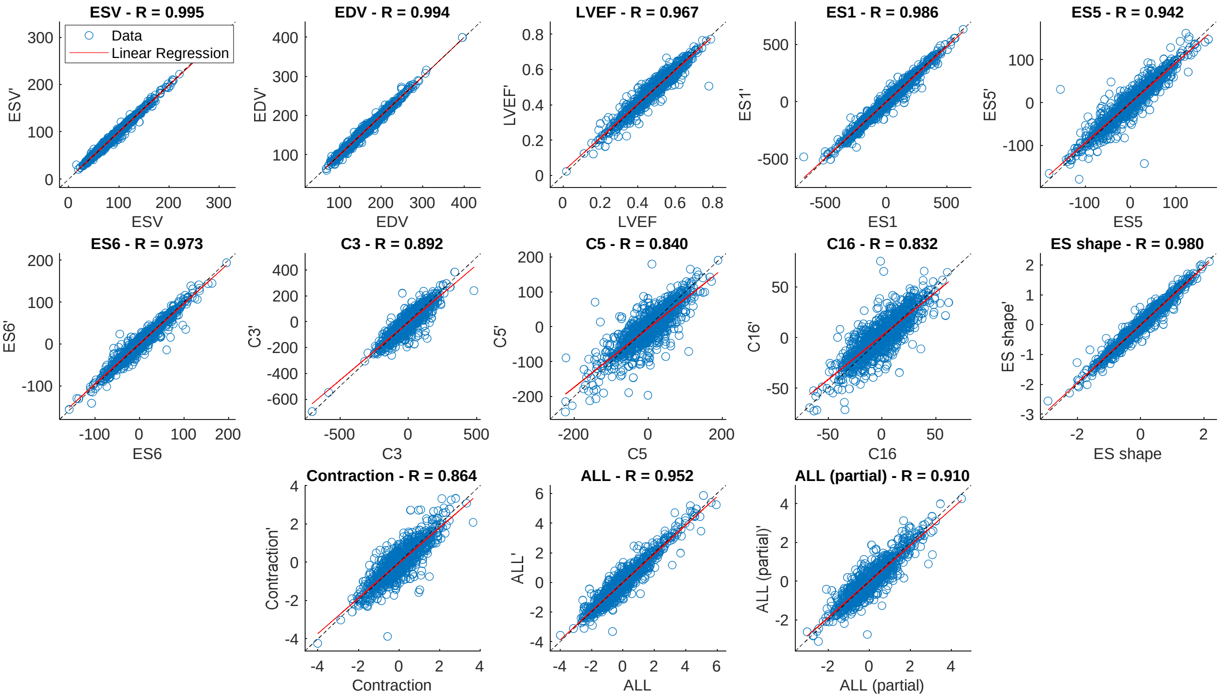

Supplemental Figure 7 – Linear correlation results of the reproducibility analysis, expressed as R score. 'x' axis corresponds to originals; 'y', reproduced. Legend reads the same as in Supplemental Table 2.

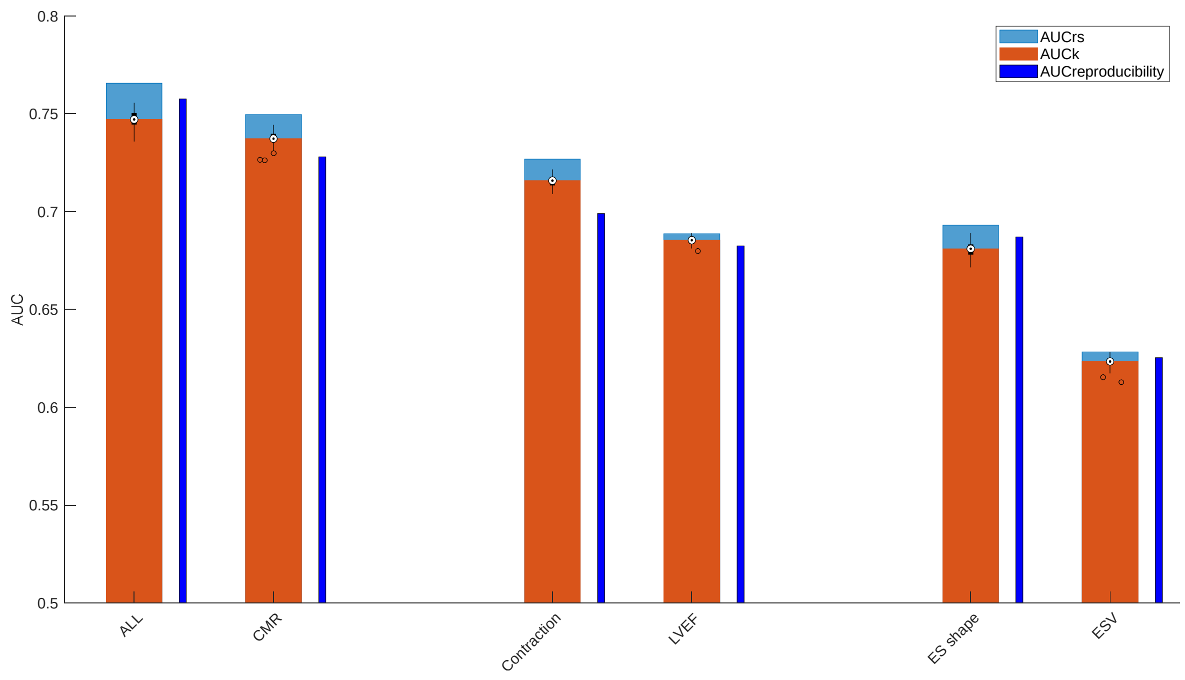

Supplemental Figure 8 – Predictive performance of ESV, LVEF, ES shape, contraction and multivariable LDA models resulting from the combination of the 3D descriptors with only CMR biomarkers (CMR) and all the variables of the study (ALL). Results are expressed as AUC re-substitution (AUCrs - light blue), AUC 10-fold cross-validated computed for 100 random data splits (AUCk - mean in orange, distribution in black) and AUC resulting from the reproducibility analysis (AUCreproducibility - dark blue). From any of the AUC metrics it can be concluded that the proposed 3D disentanglement of ESV and LVEF not only outperforms the stand-alone versions, but it also contributes to a significant overall risk management improvement in a multivariable setting including CMR markers, cardiovascular risk factors and basic patient characteristics.

SUPPLEMENTAL RESULTS: End Points Prediction

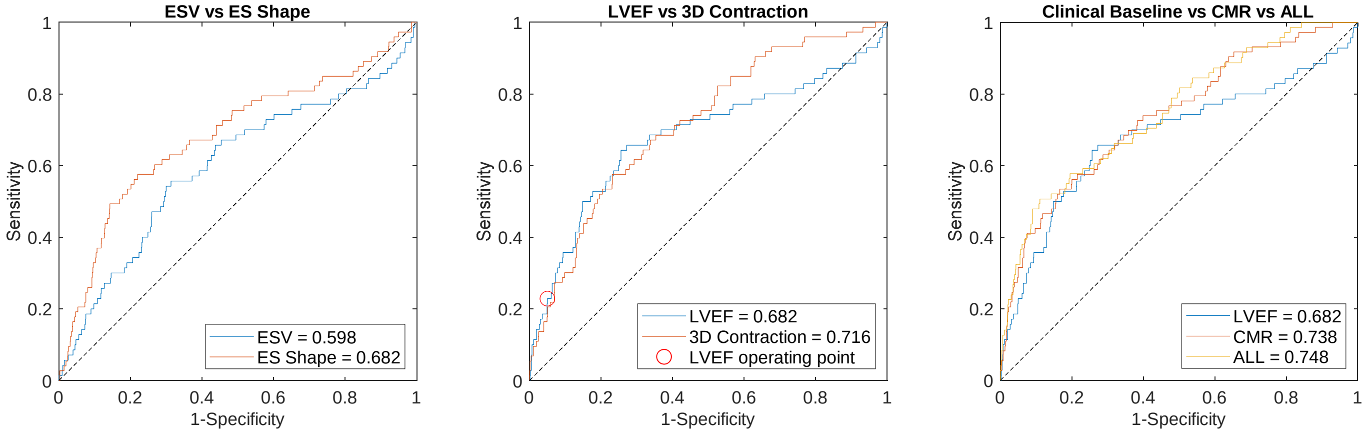

Supplemental Figure 9 – Receiver-Operator Characteristic (ROC) curves illustrating the AUC differences in MACE prediction. The curves are obtained applying leave-one-out cross-validation, and the resulting AUCs are reported in the figure legends. The interpretation of the ROC in terms of specificity and sensitivity is explained in detail in Supplemental Figure 12. Preventive treatment for patients at risk usually involves costly and invasive procedures (e.g. implantation of CRT devices). In such scenario, classifiers are typically operated at high sensitivity (i.e. ensuring that the patients predicted as at risk are indeed likely to suffer from MACE and, thus, minimizing false positives). This is the region where our proposed multivariable models most outperform the clinical baselines. **LEFT**: ESV vs ES shape. **MIDDLE**: LVEF vs 3D contraction. The operation point of the LVEF curve based on the threshold indicated in the clinical guidelines (0.35 LVEF) is illustrated (red circle). **RIGHT**: Best performing clinical baseline, i.e., LVEF vs the proposed multivariable models, combining the novel 3D descriptors with only CMR biomarkers (CMR) and with all the variables of the study (ALL).

Supplemental Table 3 – Multivariable Models: Endpoint prediction.

MODEL	LINEAR SELECTION	AUC_K	AUC_{RS}	COX SELECTION	C-$index_k$	C-$index_{RS}$
CMR Baseline	ESV, EDV	0.701 (0.699 - 0.702)	0.708	ESV, EDV	0.688 (0.687 - 0.690)	0.693
CMR Substitution	ES1, ES5, ES6, C3, C5, C16	0.729 (0.727 - 0.732)	0.748	ES1, ES5, ES6, C3, C5, C16	0.715 (0.713 - 0.718)	0.730
CMR	ESV, EDV, C5, C16	0.738 (0.736 - 0.740)	0.750	ESV, EDV, C5, C16	0.728 (0.727 - 0.730)	0.736
ALL Baseline	ESV, EDV, Age, Killip	0.728 (0.725 - 0.731)	0.745	ESV, EDV, Age, Killip	0.715 (0.713 - 0.718)	0.726
ALL Substitution	Age, Killip, BSA, ES1, C5, C16	0.757 (0.755 - 0.760)	0.776	Age, Killip, BSA, ES1, C5, C16, Vessels	0.746 (0.744 - 0.749)	0.764
ALL	ESV, EDV, Age, Killip, C5, C16	0.747 (0.745 - 0.749)	0.766	ESV, EDV, Age, C5, C16	0.741 (0.739 - 0.744)	0.753

Backward stepwise LDA and Cox results of the multivariable models including only CMR biomarkers (CMR) and all the variables of the study (ALL). While the baseline multivariable models exclude the novel 3D descriptors from the analysis, the substitution multivariable models do not consider the conventional imaging markers LVEF and ES. In the "CMR" and "ALL" models, both conventional and novel 3D markers are included as predictor candidates, as described in Methods. This demonstrates that ES shape and 3D contraction produce a significant overall improvement in a multivariable setting both in substitution and in combination with the conventional imaging markers. The ESV, EDV and LVEF calculated from automated volumes were considered for the analysis. The resulting significant selection of variables is reported along with the predictive performance, expressed as AUC and C-index re-substitution (RS) and 10-fold cross-validated (K), respectively. AUC_k and C-$index_k$ are presented as median (interquartile range). All differences were statistically significant (P<0.001). Killip indicates Killip class on admission; and vessel, number of diseased vessels.

Supplemental Table 4 – LDA models coefficients

MODEL	VARIABLES	LDA COEFFICIENTS	CONSTANT
ES Shape	ES1, ES5, ES6	0.42, -0.35, -0.46	-2.77
3D Contraction	C3, C5, C16	0.56, -0.53, -0.47	-2.89
CMR	ESV, EDV, C5, C16	1.76, -1.34, -0.33, -0.47	-3.07
ALL	ESV, EDV, Age, Killip, C5, C16	1.40, -0.98, 0.48, -0.34, -0.43, 0.51	-3.28

Coefficients and constant of the resulting ES shape, 3D contraction, CMR and ALL multivariable LDA models following the backward stepwise variable selection (see Methods). All the variables were normalized to zero mean and unit variance prior to LDA fitting. The performance of these models is summarized in Table 2 of the main manuscript.

Supplemental Table 5 – Cox models hazard ratios

TYPE	MODEL	VARIABLES	HR	*P* value
Clinical Baselines	ESV	ESV	1.43 (1.18 - 1.73)	< 0.001
	LVEF	LVEF	0.80 (0.74 - 0.87)	< 0.001
3D analysis	ES Shape	ES1	1.46 (1.17 - 1.83)	< 0.001
		ES5	0.73 (0.58 - 0.91)	0.005
		ES6	0.61 (0.48 - 0.78)	< 0.001
	3D Contraction	C3	1.66 (1.34 - 2.06)	< 0.001
		C5	0.63 (0.51 - 0.78)	< 0.001
		C16	0.66 (0.53 - 0.83)	< 0.001
Multivariable	CMR	ESV	4.23 (2.72 - 6.60)	< 0.001
		EDV	0.29 (0.18 - 0.48)	< 0.001
		C16	0.64 (0.51 - 0.80)	< 0.001
		C5	0.76 (0.61 - 0.95)	0.018
	ALL	ESV	3.41 (2.15 - 5.40)	< 0.001
		C16	0.65 (0.51 - 0.81)	< 0.001
		EDV	0.39 (0.23 - 0.65)	< 0.001
		Age	1.55 (1.19 - 2.02)	0.001
		C5	0.76 (0.60 - 0.95)	0.019

Hazard ratios, HR (95% confidence interval), and predictor significance, *P* value, of the selected variables in the resulting Cox models, presented in Table 2 of the main manuscript, along with their performance. All the variables were normalized to zero mean and unit variance prior to Cox analysis.

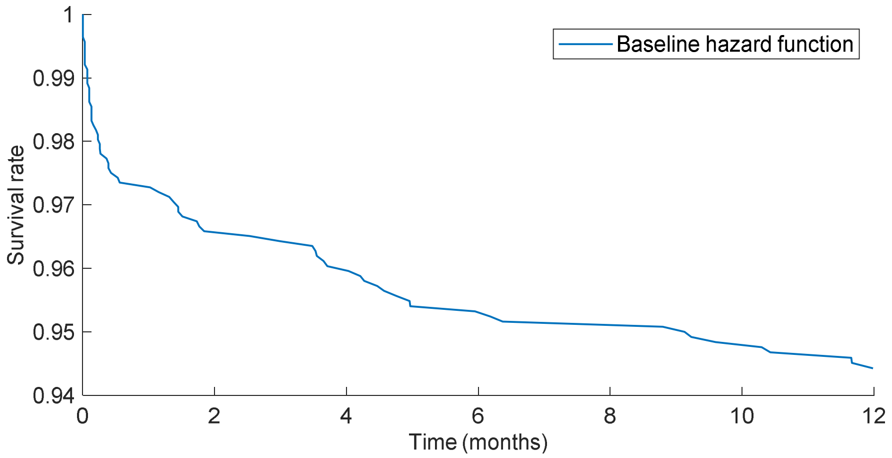

Supplemental Figure 10 – Underlying baseline hazard function

Supplemental Table 6 – Endpoint prediction using volumes from manual segmentations

MODEL	LINEAR SELECTION	AUC_K	AUC_{RS}	COX SELECTION	C-index_k	C-index_{RS}
CMR	ESV, EDV, C5, C16, ES1	0.754 (0.752 - 0.756)	0.767	ESV, EDV, C5, C16, ES1	0.737 (0.735 - 0.739)	0.748
ALL	ESV, EDV, Age, C5, C16, ES1	0.762 (0.760 - 0.765)	0.779	ESV, EDV, Age, C5, C16, ES1	0.746 (0.743 - 0.749)	0.760

Backward stepwise LDA and Cox results of the multivariable models including only CMR biomarkers (CMR) and all the variables of the study (ALL). The ESV, EDV and LVEF calculated from manual segmentations were selected for the analysis. The resulting significant selection of variables (in red, differences with respect to the selection results using automated volumes) is reported along with the predictive performance, expressed as AUC and C-index, under cross-validation (k=10) and re-substitution (RS). AUC_k and C-index_k are presented as median (interquartile range). All differences were statistically significant (*P*<0.001). Note the improvement in performance achieved by these models in comparison to the results reported in Table 2 of the main manuscript, related to the same analysis considering automated volumes rather than manual ones. This improvement could be explained by a double observer effect, where ESV and EDV are manually obtained, and a second observer (a machine) gets the contours again to find the meshes and modes.

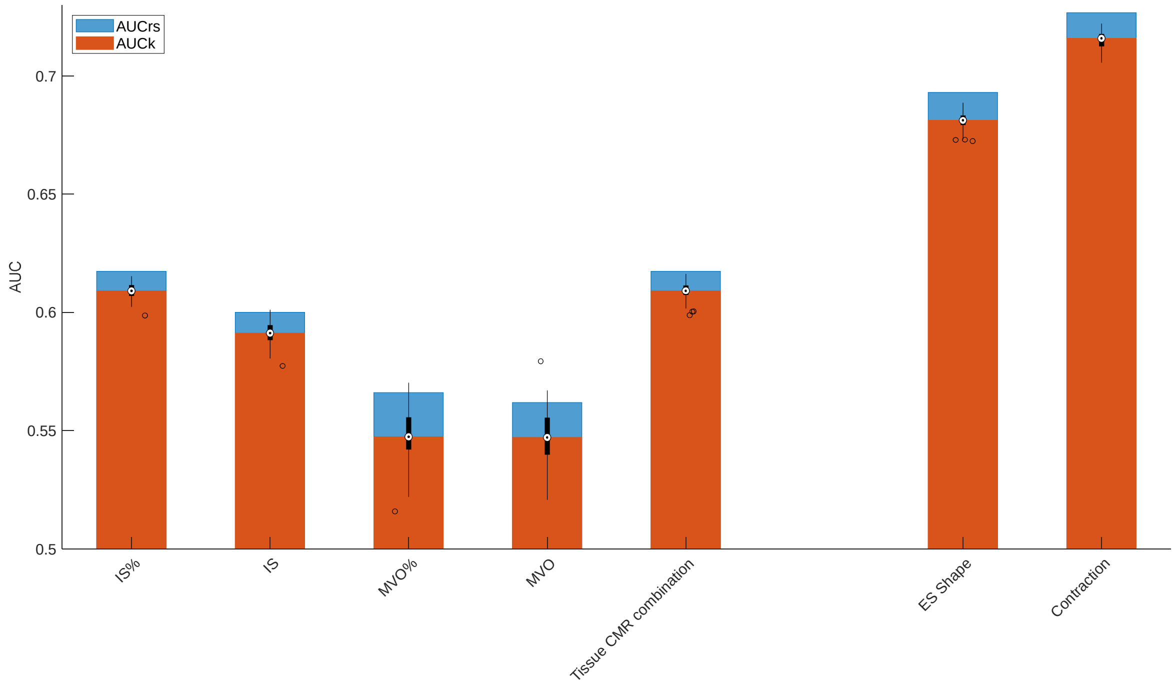

Supplemental Figure 11 – MACE prediction comparison between the proposed ES shape and 3D contraction novel markers versus the tissue CMR biomarkers. Assessment based on AUC re-substitution (AUCrs, blue) and AUC 10-cross-fold validated (AUCk, orange), repeated for 100 random data splits (dark distributions). IS indicates infarct size; IS%, infarct size to LV mass ratio; MVO, microvascular obstruction; and MVO% microvascular obstruction to LV mass ratio. Tissue CMR combination is the resulting model from the backward stepwise LDA including all the tissue CMR biomarkers. Note that this model is identical to IS%, i.e. the rest of the tissue CMR biomarkers do not add significant prognostic value. All the differences in performance between the proposed 3D biomarkers and the tissue CMR ones were significant (*P*<0.001).

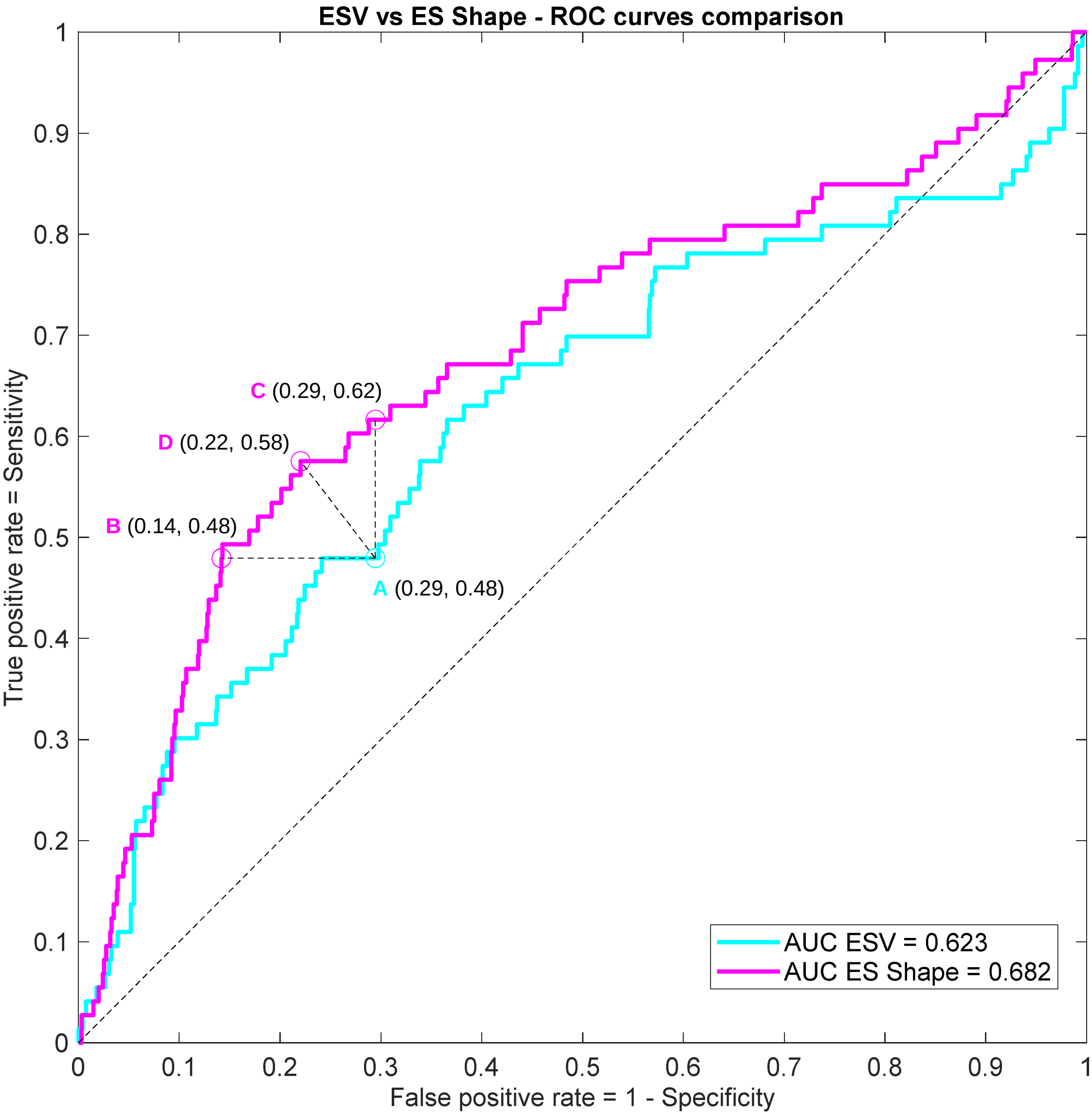

Supplemental Figure 12 – Interpretation and implications of the AUC differences in ROC prediction curves, illustrated by the ESV vs ES shape comparison. Given a certain sensitivity and specificity operating point of the ESV curve, i.e. point A, the ES shape model is able to predict MACE at the same sensitivity but reducing the false positive rate a 50% (point B), i.e., from 275 false positive MACE predictions to 133. Likewise, the ES shape model could operate at a similar false positive rate to the ESV model while improving the sensitivity by 29% (point C). Alternatively, the ES shape operating point D could be chosen for MACE prediction, with both sensitivity and specificity improved with respect to the ESV model. This demonstrates the potential for risk management improvement associated to superior AUC scores.

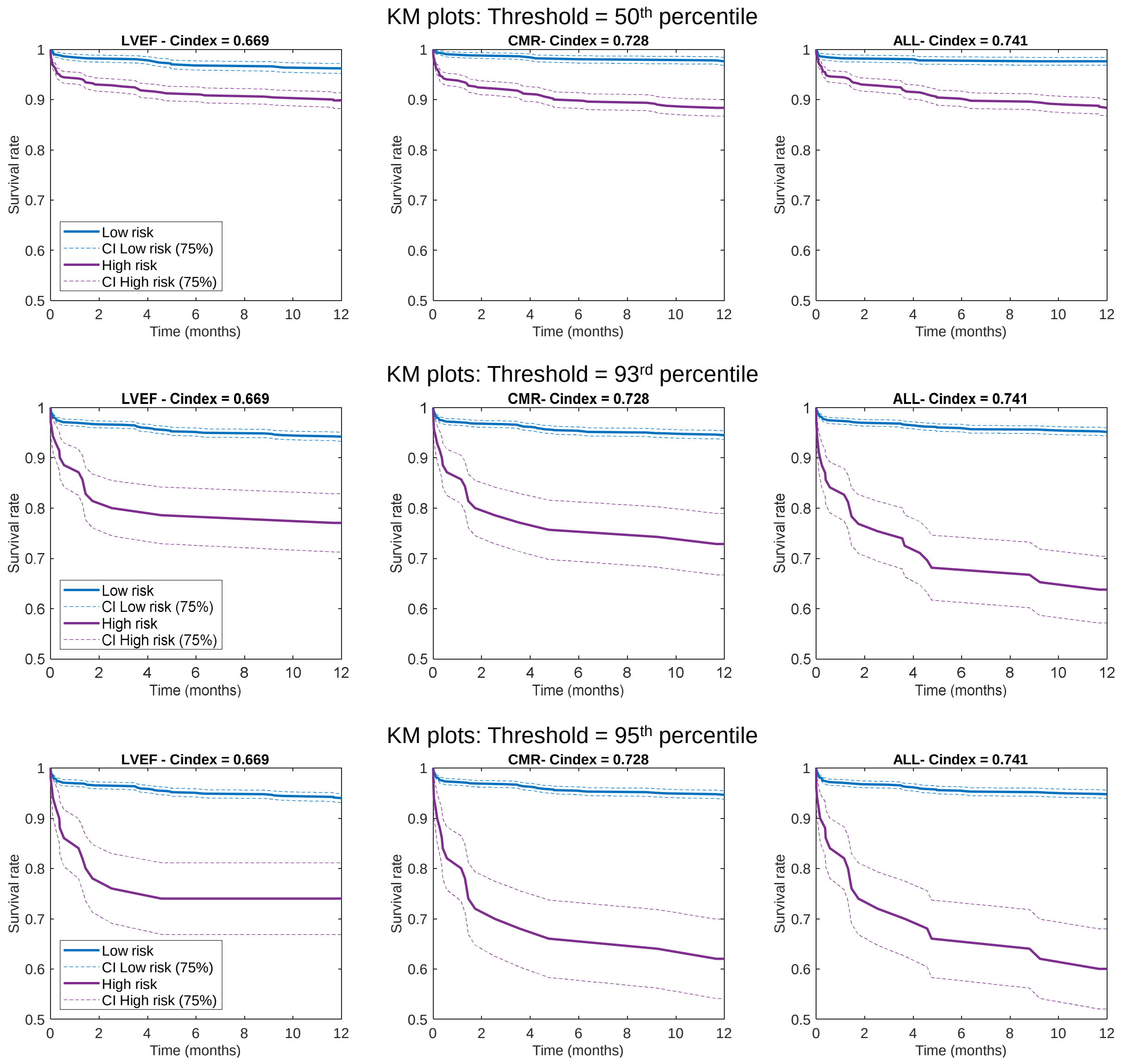

Supplemental Figure 13 – Kaplan–Meier (KM) curve estimates for the high (purple) and low (blue) risk groups according to the best performing clinical baseline model, LVEF (**left**), and our proposed multivariable models, CMR (**middle**) and ALL (**right**). The KM plots are calculated as follows: (1) once trained, the Cox model is used to predict the patient scores; (2) the patients are then sorted by score, and the high and low risk groups are separated based on a threshold (a percentile); and, finally, (3) the KM curves, along with the confidence intervals, are estimated for each group of patients based on their initial survival data. This means that the KM curves illustrate survival at a single operating point, determined by the threshold selection. To illustrate this, 3 operating thresholds are considered: median percentile (**top**), 93rd percentile (**middle** – the operating point of a theoretical classifier that matches the 7% MACE incidence) and 95th percentile (**bottom** – operating point to increase sensitivity). Given our MACE incidence, the high and low risk KM curves are necessarily similar when operating at the median percentile, since survival is the main trend in both groups. As we move towards higher percentiles, the differences between high/low groups increase. The CMR model performs only slightly better than LVEF at 93rd percentile, but it is as good as the ALL model at 95th percentile. The assessment is biased to the threshold selection. This is why we prefer to establish comparisons based on C-indices (herein reported) and the ROC curves (Supplemental Figure 9), which illustrate performance at any of the sensitivity-specificity operating points.

SUPPLEMENTAL RESULTS: ES Shape Modes

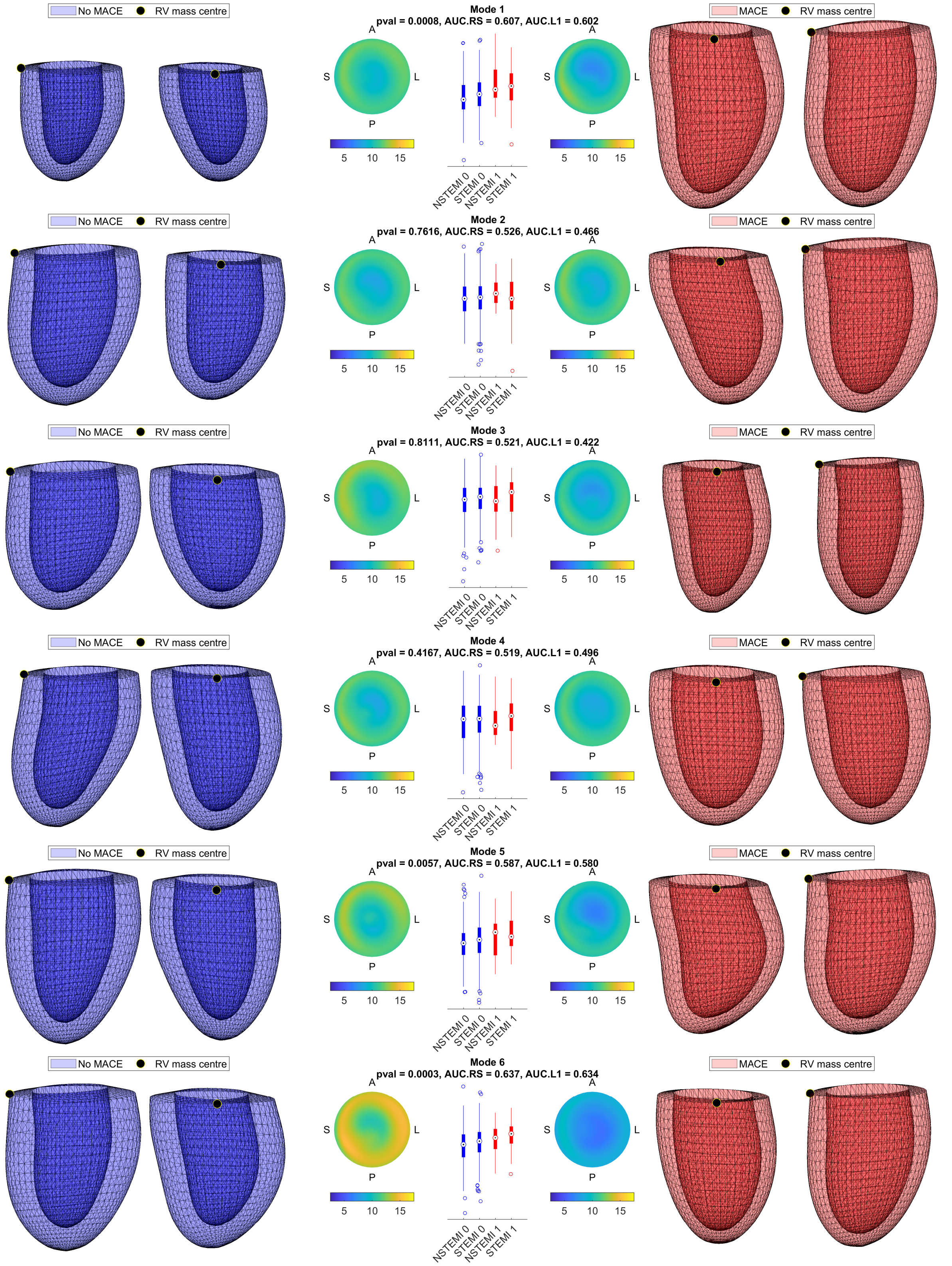

Continued in next page

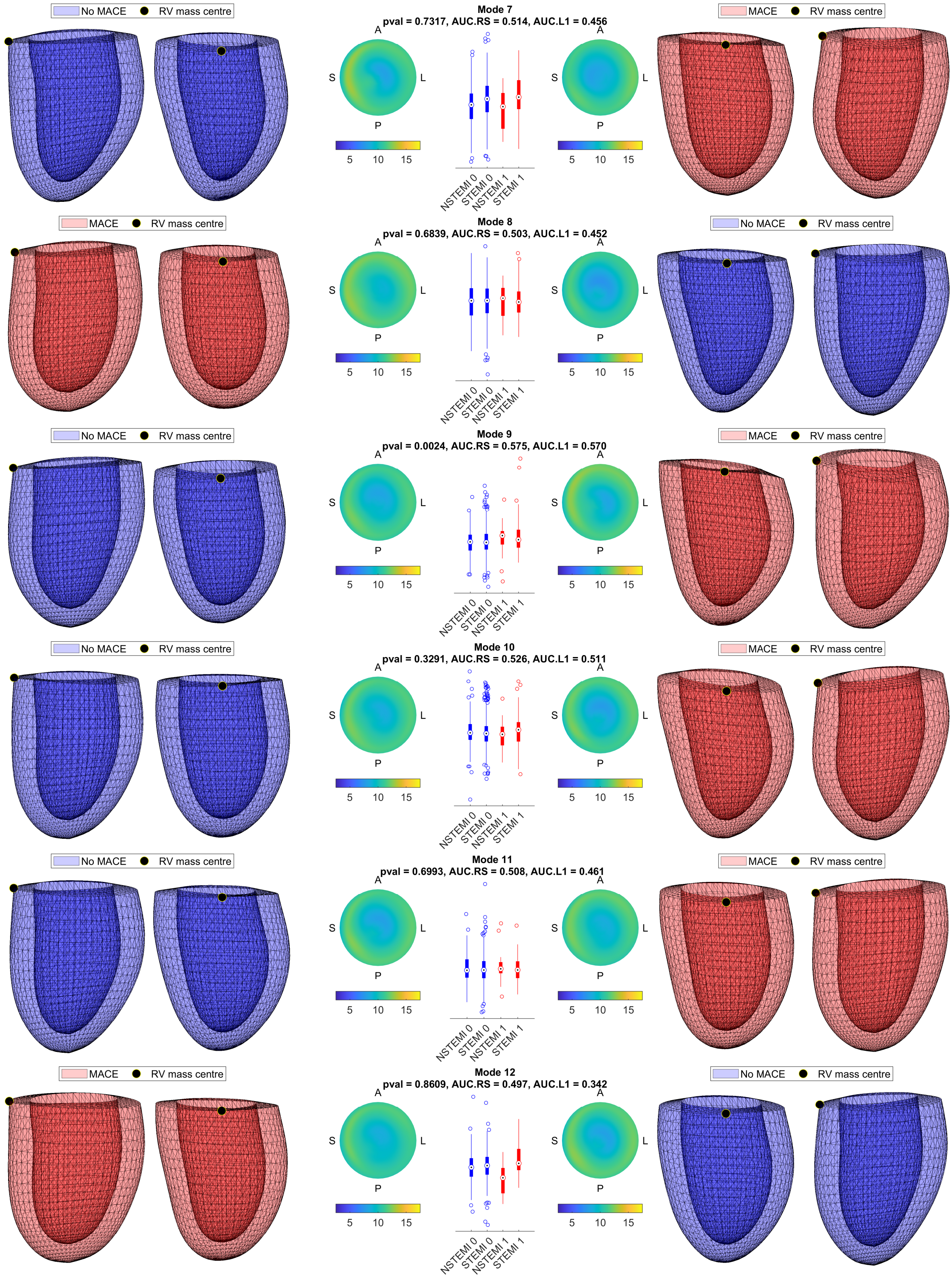

Supplemental Figure 14 – ES shape main modes of variation accounting for 95% of the population variance. The meshes are presented in anterior and septal views and in the form of thickness maps on a circumferential polar plot as in the 17-segments AHA model. The MACE (red, class 1) and No MACE (blue, class 0) representations correspond to the 5th and 95th percentiles in the mode direction. *P* value and re-substitution and leave-one-out AUCs are presented along with the MACE and no MACE distributions, further stratified into infarct aetiology (STEMI and NSTEMI).

SUPPLEMENTAL RESULTS: 3D Contraction Modes

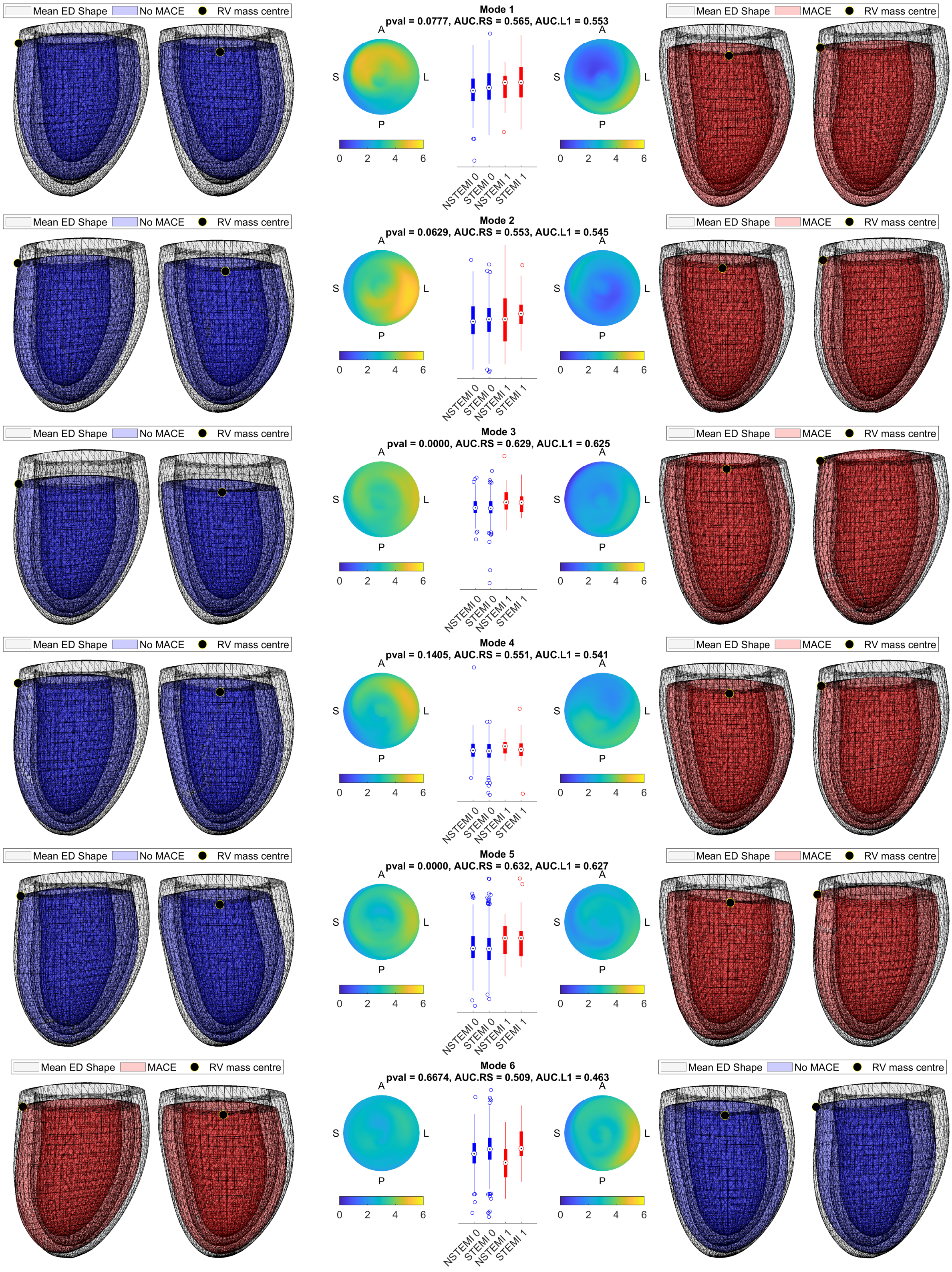

Continued in next page

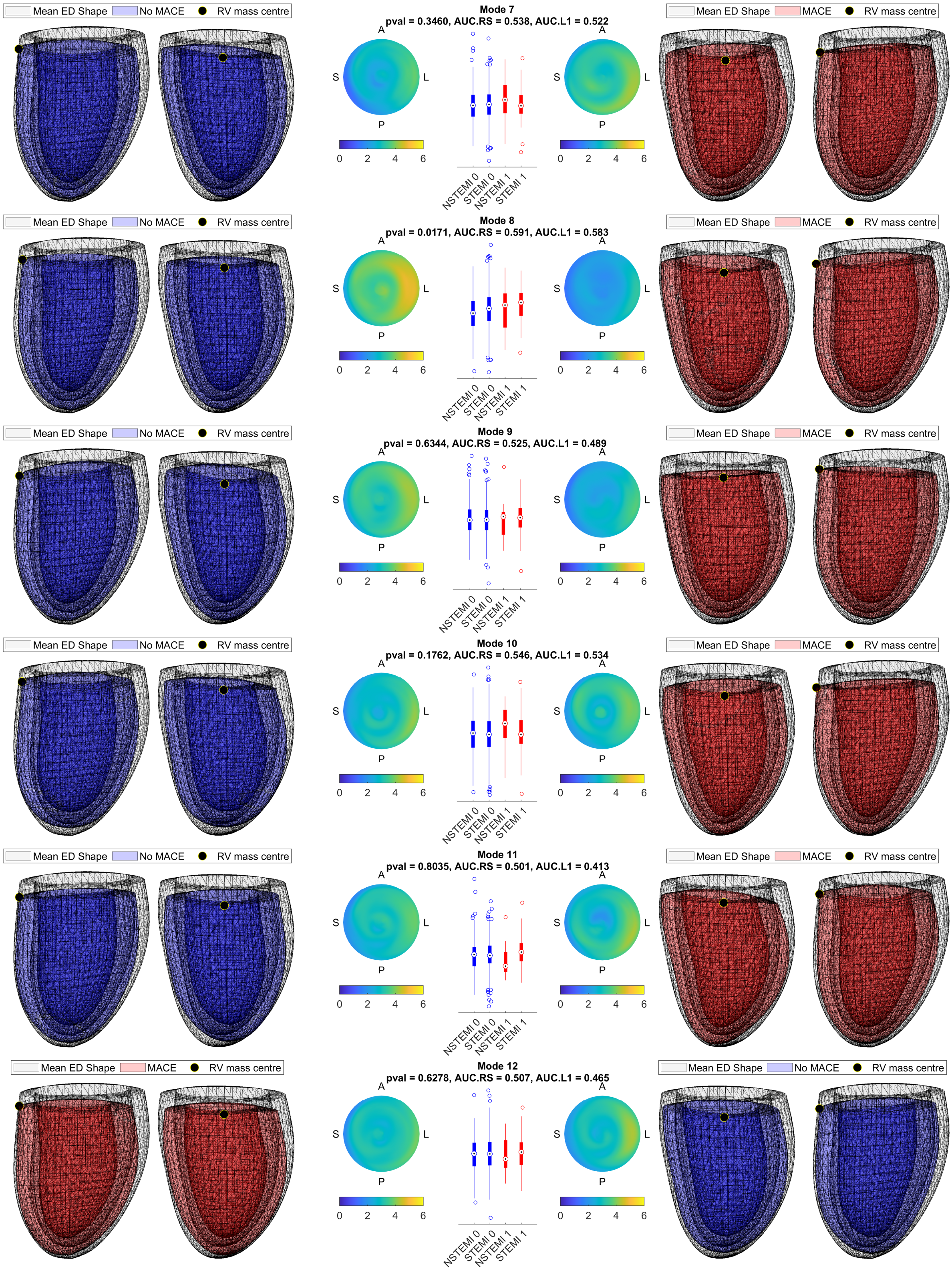

Continued in next page

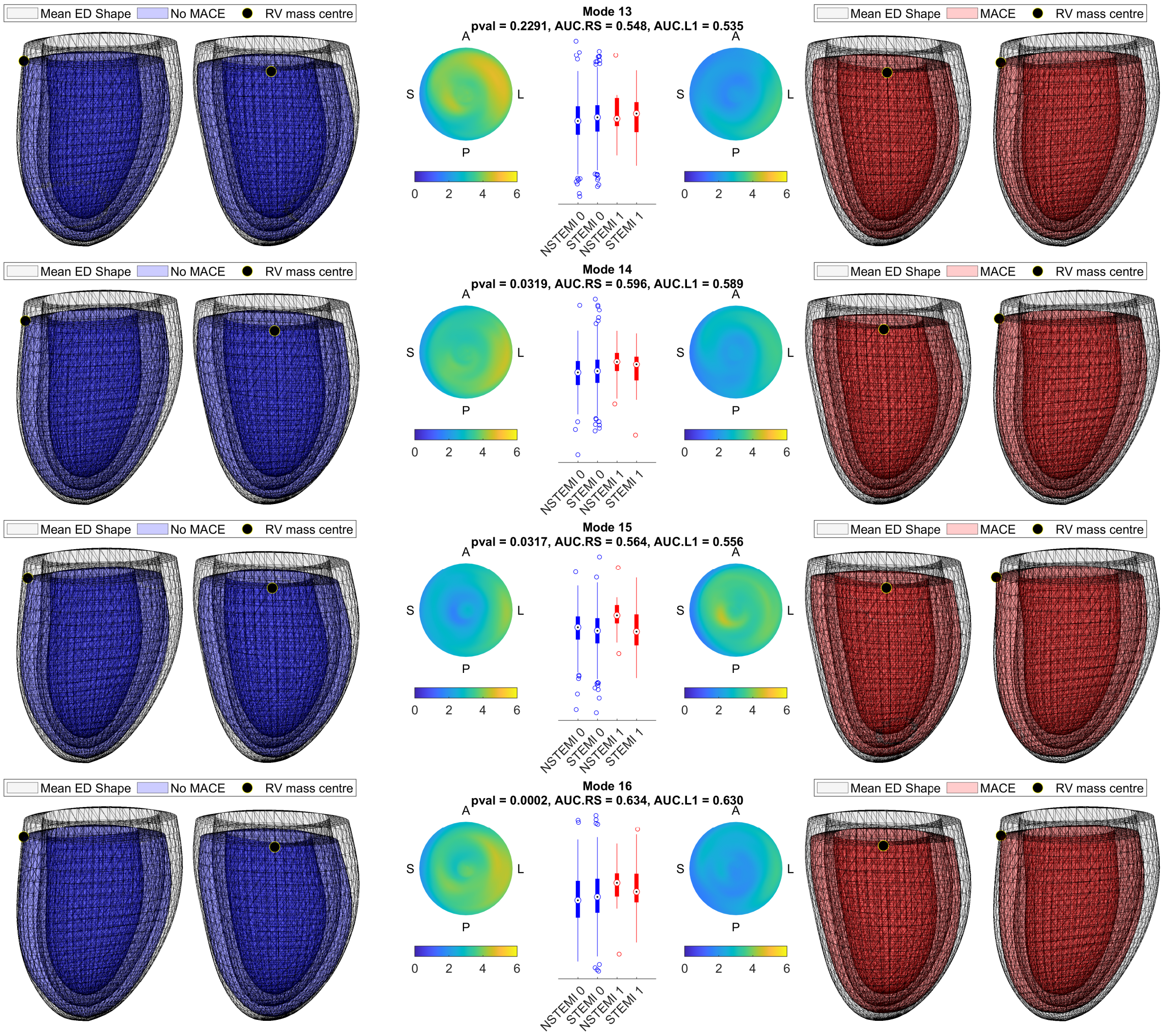

Supplemental Figure 15 – LV contraction modes 1 to 16, accounting for 93.2% of the population variance. The meshes are presented in anterior and septal views and in the form of differential thickness maps (ED minus ES thickness) on a circumferential polar plot as in the AHA model. To facilitate comparisons, the contractions are applied on the mean ED shape and visualized as resulting ES shapes. The MACE (red, class 1) and No MACE (blue, class 0) representations correspond to the 5th and 95th percentiles in the mode direction. *P* value and re-substitution and leave-one-out AUCs are presented along with the MACE and no MACE distributions, further stratified into infarct aetiology (STEMI and NSTEMI). The mean ED shape (transparent surface) is included as reference.

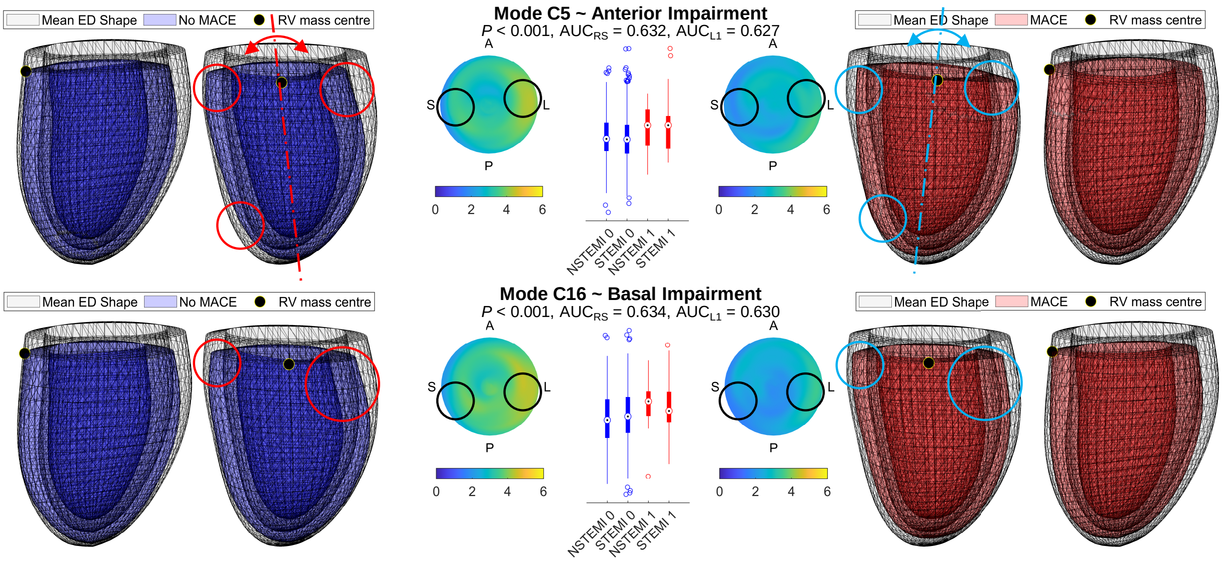

Supplemental Figure 16 – Repetition of the contraction modes 5 and 16, shown in the previous figure and proven relevant to AMI prognosis (see main manuscript), highlighting the differences between the MACE and No MACE contractions. While C5 shows an anterior impairment, the main difference in C16 is the basal impairment between the 2 configurations, particularly visible in the anterior wall, but also affecting the posterior one. A difference in contraction thickening of 4 vs 2 mm is also observed in the lateral and septal walls as a secondary change.

SUPPLEMENTAL RESULTS: Subgroup Analysis

The proposed methodology has superior prognostic value for patients with decreased LV function (LVEF<35%), identified as high-risk group, in accordance to the literature (3) (see Supplemental Figure 17). With respect to infarct aetiology, the method is more predictive for the NSTEMI population (AUC of 0.79 vs 0.76, respectively, applying 'ALL' multivariable model – see Methods) despite the study being biased to STEMI patients (see Results). While mechanistic explanations are to be further explored, clinical validity for both types can be argued. This is also evidenced in the independent testing experiment, where models trained on only STEMI patients are suitable to NSTEMI ones (see Supplemental Results: Independent Testing). Additional investigations show that ES5 and ES6 are significantly higher for the STEMI population, i.e. thinner ES shapes (as result of larger infarct size, P<0.001, in agreement with (5)) and higher anterior curvatures (LAD infarct (9)), respectively. Finally, while C16 is not relevant to infarct type, it is significantly higher for the NSTEMI MACE subgroups, which in turn may explain its predictive power.

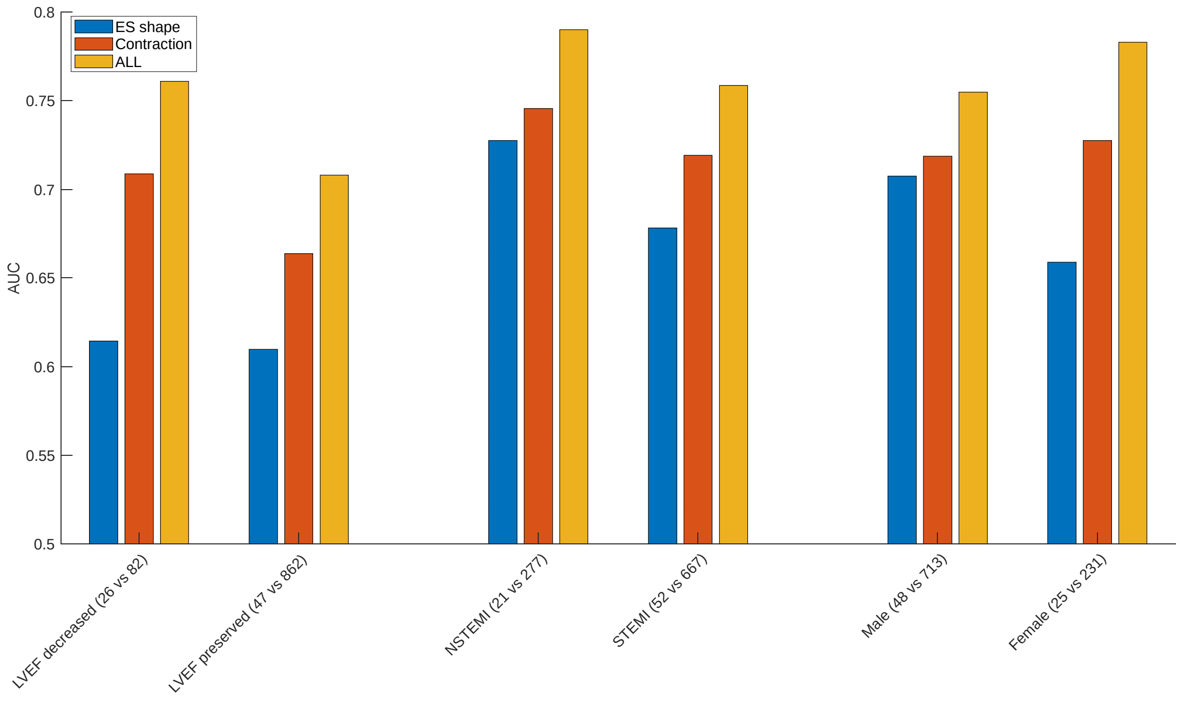

Supplemental Figure 17 – AUC endpoints prediction results stratifying by gender, infarct aetiology and LVEF (threshold: 0.35); and applying the 'ES shape', '3D contraction' and 'ALL' LDA models resulting from the backward stepwise analysis (See Table 2 and Supplemental Figure 10). The labels are presented as subgroup (MACE vs No MACE cases).

SUPPLEMENTAL RESULTS: Modes Correlation Analysis

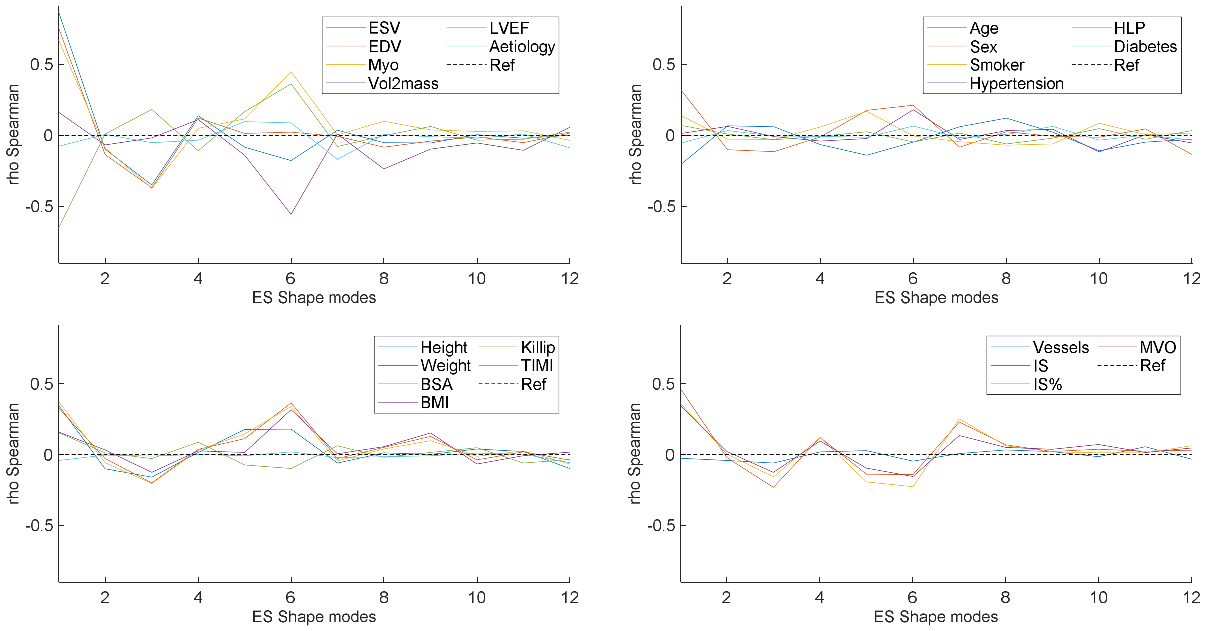

Supplemental Figure 18 – Spearman correlation between the first 12 ES shape modes of variation and all the variables included in the study. Myo indicates LV myocardial mass; Vol2mass, EDV to LV mass ratio; Aetiology, infarct aetiology (STEMI vs NSTEMI); HLP, Hyperlipoproteinemia; BSA, body surface area; BMI, body mass index; Killip, Killip class on admission; TIMI, TIMI flow grade post-PCI; Vessels, Nr. of diseased vessels; IS, infarct size; IS% infarct size to LV mass ratio; MVO, microvascular obstruction. Ref is the reference line of zero correlation.

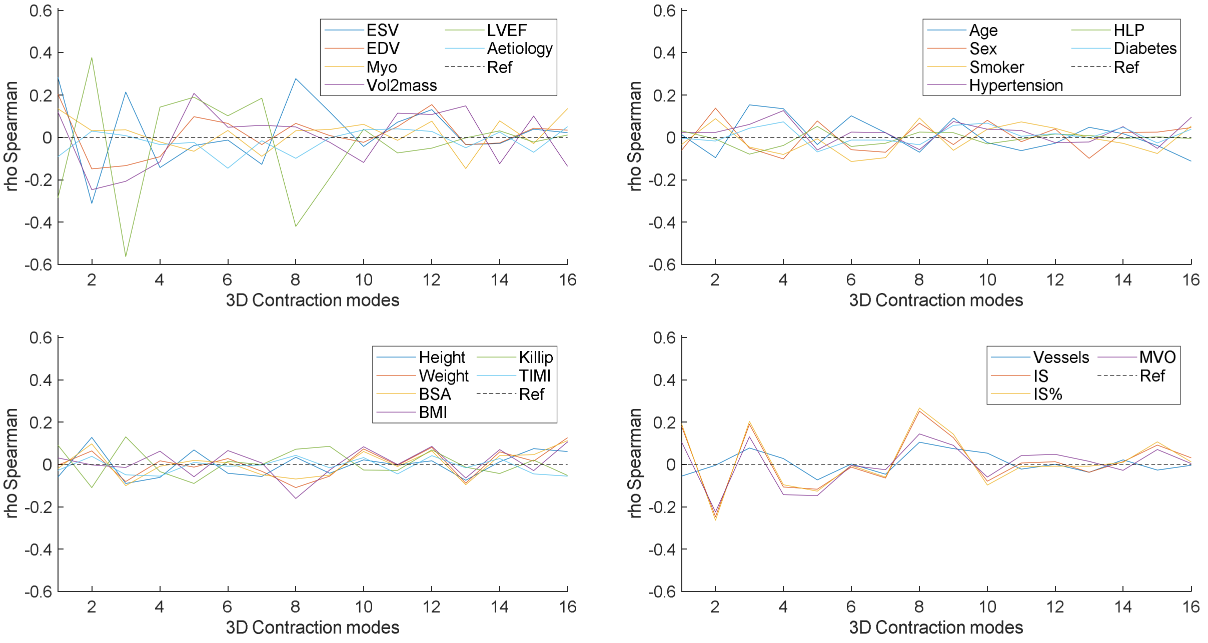

Supplemental Figure 19 – Spearman correlation between the first 16 3D contraction modes of variation and all the variables included in the study. Legend reads the same as in Supplemental Figure 18 above.

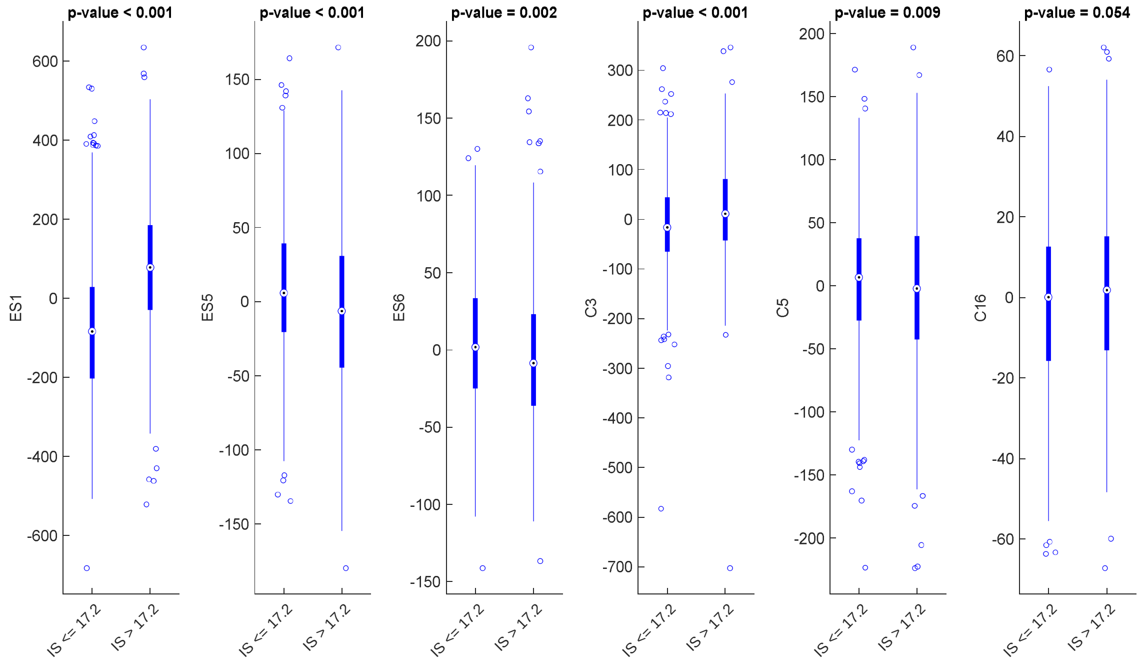

Supplemental Figure 20 – Distribution and significance of the identified risk-related shape and contraction variations (ES1, ES5, ES6, C3, C5 and C16) in stratifying **IS** (infarct size) into low/high myocardial damage using the median value of the AMI population as threshold (17.2 mL).

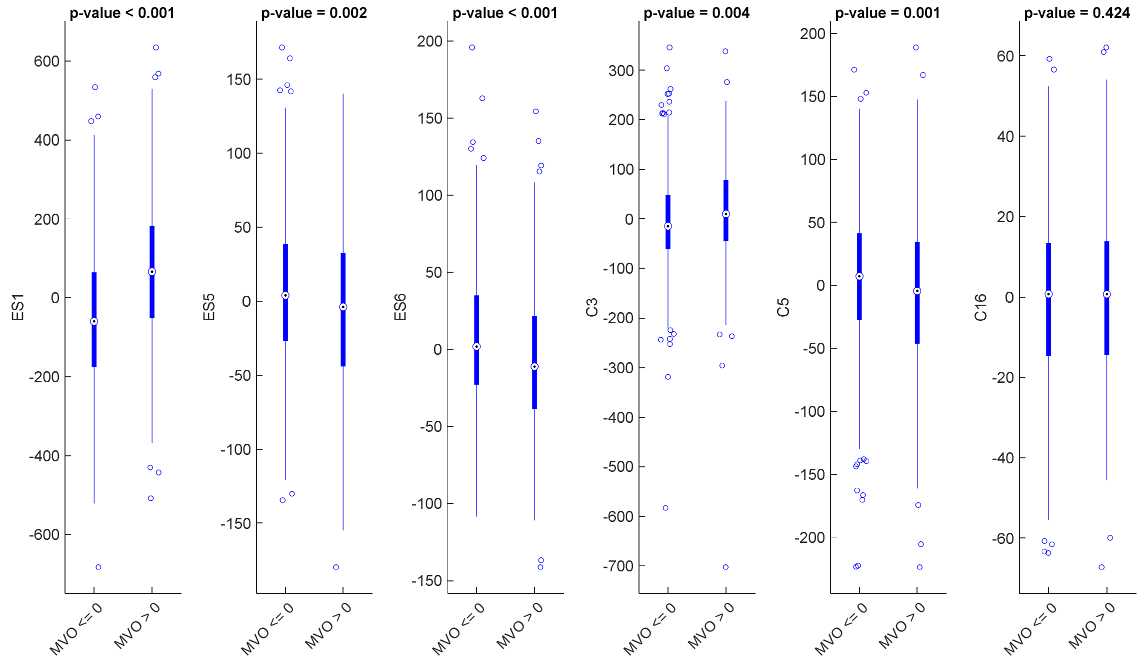

Supplemental Figure 21 – Distribution and significance of the identified risk-related shape and contraction variations (ES1, ES5, ES6, C3, C5 and C16) in stratifying **MVO** (microvascular obstruction) into low/high damage using the median value of the AMI population as threshold (0 mL, that is, intact microvasculature.
